# Differences between microhabitat and broad-scale patterns of niche evolution in terrestrial salamanders

**DOI:** 10.1038/s41598-018-28796-x

**Published:** 2018-07-12

**Authors:** Gentile Francesco Ficetola, Enrico Lunghi, Claudia Canedoli, Emilio Padoa-Schioppa, Roberta Pennati, Raoul Manenti

**Affiliations:** 1 University Grenoble Alpes, Laboratoire d’Écologie Alpine (LECA), F-38000 Grenoble, France; 2 CNRS, Laboratoire d’Écologie Alpine (LECA), F-38000 Grenoble, France; 3 Department of Earth and Environmental Sciences, Università degli Studi di Milano-Bicocca, Piazza della Scienza 1, 20126 Milano, Italy; 4 Department of Environmental Science and Policy, Università degli Studi di Milano, Via Celoria 26, 20133 Milano, Italy; 5 Universität Trier Fachbereich VI, Campus I, Gebäude N Universitätsring 15, 54286 Trier, Germany; 6 Natural Oasis, Via di Galceti 141, 59100 Prato, Italy; 7 Natural History Museum of the University of Florence, Section of Zoology “La Specola”, Via Romana 17, 50125 Firenze, Italy

## Abstract

The extent to which closely related species share similar niches remains highly debated. Ecological niches are increasingly analysed by combining distribution records with broad-scale climatic variables, but interactions between species and their environment often occur at fine scales. The idea that macroscale analyses correctly represent fine-scale processes relies on the assumption that average climatic variables are meaningful predictors of processes determining species persistence, but tests of this hypothesis are scarce. We compared broad- and fine-scale (microhabitat) approaches by analyzing the niches of European plethodontid salamanders. Both the microhabitat and the macroecological approaches identified niche differences among species, but the correspondence between micro- and macroecological niches was weak. When exploring niche evolution, the macroecological approach suggested a close relationship between niche and phylogenetic history, but this relationship did not emerge in fine-scale analyses. The apparent pattern of niche evolution emerging in broad-scale analyses likely was the by-product of related species having closely adjacent ranges. The environment actually experienced by most of animals is more heterogeneous than what is apparent from macro-scale predictors, and a better combination between macroecological and fine-grained data may be a key to obtain robust ecological generalizations.

## Introduction

The idea that phylogenetically related species also tend to be ecologically similar has intrigued researchers since Darwin’s Origin of Species^1^. Phylogenetic conservatism is the tendency of closely related species to be more similar than expected under randomness^1,2^. Phylogenetic signal is often observed for morphological and life history traits (e.g. refs^1–4^), and has also been detected for traits representing species niche, such as eco-physiological features, climatic niche, diet and habitat^1,5^. Nevertheless, signal for niche traits is not ubiquitous, as many studies have actually found a high evolutionary lability of realized niches^1,5^. There is thus a growing interest in the study of phylogenetic signal of niches, and of the conditions and traits for which effects of phylogenetic signal on niche are stronger or can be better detected^1,5^.

The evolution of niches is often analysed through a broad-scale (bioclimatic) approach, i.e. by combining species distribution data with coarse-resolution, ‘scenopoetic’ variables^5^. These macroecological approaches have had increasing appeal given the availability of broad-scale information (e.g. species distribution data, climatic information, environmental data from remote sensing, phylogenies), and the impressive progress of ecological informatics^6^. The broad geographical scale of these studies is both a strength and a limitation. Working over macro-scales allows drawing general patterns that are hardly recovered using local analyses, but the data available over broad scales generally have a coarse resolution. For instance, most of analyses of relationships between animals and climate are performed at scales that are ~10,000 times larger than the study organisms^6,7^. However, it is widely recognized that species distributions are the product of multi-scalar processes, and many interactions between species and the environment occur at fine scales^8,9^. Thus, abiotic conditions actually experienced by individuals do not necessarily correspond to such macro-predictors^10–12^, and bioclimatic predictors often are just surrogates of the fine-scale environmental features actually experienced by individuals^11^.

Until now, many studies have implicitly assumed that broad-scale variables are meaningful predictors of the parameters influencing species (mean field approximation)^13^, without comparing the effects of micro- and macro-scale conditions. In order to assess how climate determines the distribution of species we need testing the appropriateness of the mean field approximation, and thus comparing the outcome of micro- and macroclimate analyses^13^. Such comparison can be performed using statistical downscaling^11,14,15^ or explicit modelling of microclimate^16^, but these approaches suffer some limitations^15^, and do not empirically assess the actual microclimates exploited by organisms.

**Figure 1:**
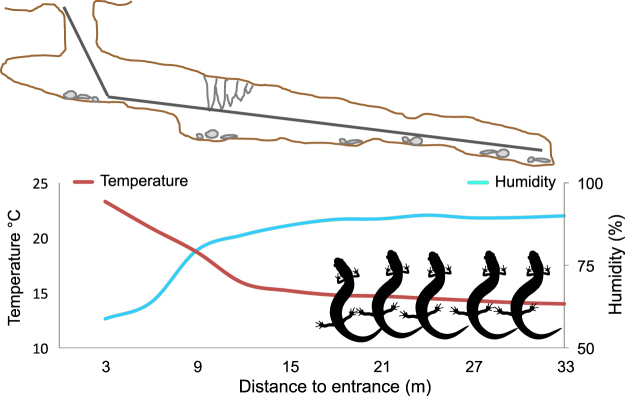
How microhabitat selection can mirror habitat selection experiments. At increasing depths, temperature decreases and humidity increases: salamanders are only found when conditions are within the species range. The figure represents the microhabitat and salamander distribution actually observed in the cave “*Brecca su Fenugu”* (39°42′N, 9°25′E). The salamander image is by N. Sinegina. The image was obtained from (http://www.supercoloring.com/silhouettes/salamander) under a Creative Commons Attribution-Share Alike 4.0 Licence. https://creativecommons.org/licenses/by-sa/4.0.

Alternatively, the comparison can be performed using microclimate data from real observations^10^. Microhabitat selection and thermoregulation through behaviour are major processes allowing animals to maintain body conditions within their physiological limits, i.e. within the range of conditions imposed by the fundamental niche of the species^12^. Microhabitat selection by species in the wild can provide accurate data on species requirements, thus allowing us to draw measures of species niche, with a rationale analogous to analyses of operative temperature^12^ or to habitat preference experiments in which organisms are exposed to a variety of environmental conditions and can select those within their suitability range (Fig. 1; see e.g.^17^). Bioclimatic and microhabitat data can provide insights about different aspects of species niches. Hierarchical approaches, integrating analyses at multiple levels, can thus greatly enhance understanding of niches and help to evaluate under which conditions the different approaches are most appropriate^18,19^, but there are few multi-scalar analyses (for examples, see^9,19^).

Terrestrial salamanders have been a frequent focus of analyses of bioclimatic niche. Niche analyses have been used to infer distribution changes and declines caused by climate change, to identify broad-scale drivers of biodiversity patterns, to analyse niche evolution in a phylogenetic context and even as a tool to describe new species (e.g.)^19–23^. In this study we analysed niches of eight species of terrestrial salamanders (genus *Hydromantes*, subgenera *Speleomantes* and *Atylodes*; see Wake)^24^ using the microhabitat selection and bioclimatic approaches, and assessed the phylogenetic signal of niches with the two approaches. Despite being sometimes named “cave salamanders”, these are not true cave-dwelling organisms: underground environments just are the habitats where salamander detection is easiest^25^.

European terrestrial salamanders are an interesting group for niche analyses. First, salamanders have superficial activity during cool and wet periods (from autumn to spring), but move to underground environments during summer, when external conditions would be too harsh (e.g., dry, hot). In these environments, they select sectors having microclimatic features within their physiological limits (Fig. 1; see Methods). Their microhabitat selection is similar to what is done in habitat preference experiments, in which organisms are placed in a gradient where they select environmental conditions within their suitability range^17^, and is thus particularly appropriate to identify the tolerance of species. Actually, previous analyses have shown that microhabitat selection provides reliable information on the operative conditions of individuals, thus allowing a good characterization of species biophysical niches^26^. Second, the features of underground habitats are different but strongly dependent on conditions outside the cave (epigean). For instance, far from the surface, the mean temperature approximately corresponds to the average local temperature of the atmosphere, and underground conditions are heavily influenced by epigean variation of temperature and precipitation^27–29^. Underground environments are not a unique case, as there are many environments in which microclimate might be imperfectly modelled by macroclimate, such as streams, ponds, forests with dense understory and topographically complex landscapes^10,14,16,30,31^, thus insights of our analyses can be relevant for many species and habitats. Finally, the fauna living underground and in the soil is rarely investigated by macroecological studies^6^, even though it includes a major proportion of terrestrial biodiversity.

We analysed the niche of salamander species using both a fine-grained (microhabitat, representing the operative conditions actually experienced by individuals) and a broad-scale perspective (i.e. combining presence localities with broad-scale bioclimatic variables). We tested to what extent information on niche features and evolution is conserved between these two scales of analysis, and identified the geographical and evolutionary factors determining the mismatch between fine-grained and coarse-grained analyses of niche evolution.

## Results

**Figure 2:**
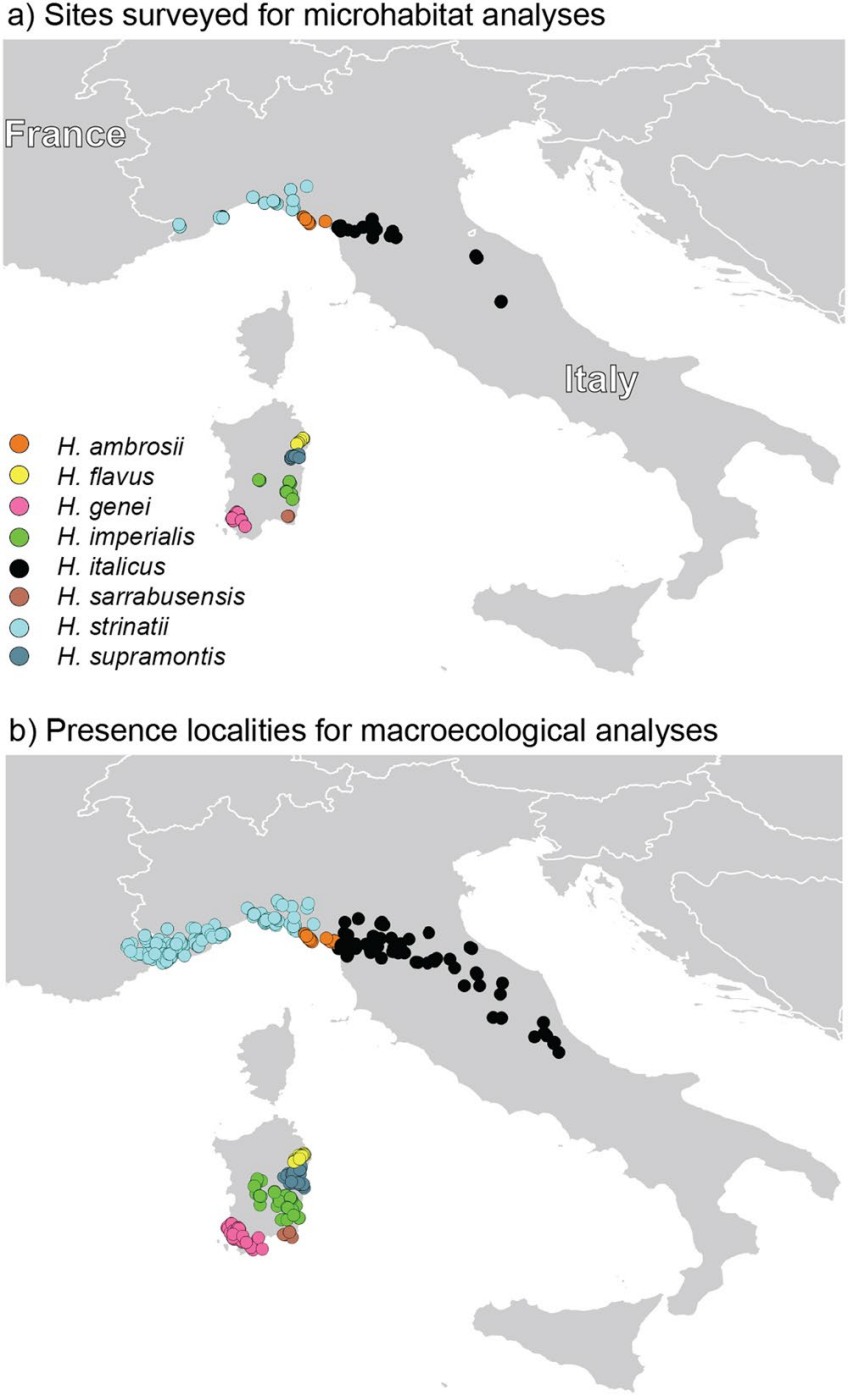
Distribution of (**a**) caves sampled for the microhabitat analyses; (**b**) presence localities used for the broad scale, macroecological analyses. The map was created using QGis 2.18 (www.qgis.org).

In field surveys, we detected >2700 salamanders in 521 out of the 1251 cave sectors; the number of sectors in which we detected salamanders was heterogeneous among species (Table 1, Fig. 2a).

**Table 1:** Caves and cave sectors sampled for the microhabitat analyses, and presence localities used for the bioclimatic analyses.

Species	Microhabitat analyses				Bioclimatic analyses
	*N* caves surveyed	*N* sectors	*N* sectors with presence	*N* individuals observed	*N* presence localities
*Hydromantes ambrosii*	40	172	91	596	65*
*H. flavus*	33	69	22	65	42
*H. genei*	29	183	66	257	54
*H. imperialis*	27	223	98	807	60
*H. italicus*	57	245	84	322	152*
*H. sarrabusensis*	8	12	5	83	10
*H. strinatii*	61	228	123	505	177
*H. supramontis*	23	119	32	119	37

Surveys covered the whole cave. Very deep caves were explored for >50 m after the detection of the deepest salamander, but very deep sectors are rarely occupied because they are difficult to reach. Therefore, to avoid an excessive number of sectors without salamanders, in analyses we only considered until the first empty sector after the last salamander.

^*^Localities within the hybrid zone between *H. ambrosii* and *H. italicus* were excluded from analyses.

### Niche analyses at the microhabitat level

**Figure 3:**
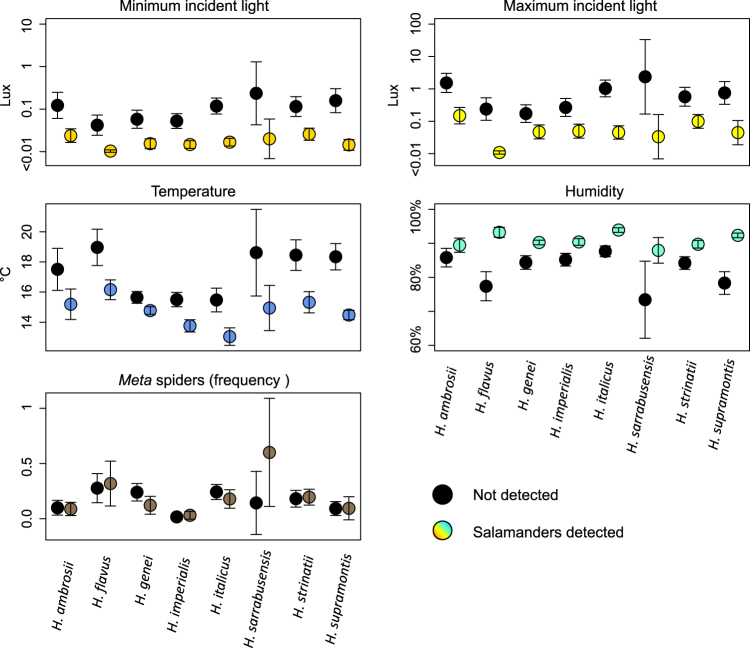
Microhabitat conditions in cave sectors where salamanders were detected (coloured dots) or undetected (black dots). Dots represent the mean conditions of occupied/unoccupied sectors; error bars are twice the standard errors.

Relationships between species presence and abiotic variables were similar across the eight salamander species. All species were significantly associated with the sectors having highest humidity, lowest temperature, and lack of light. Relationships with spiders were generally weak (Fig. 3, Table 2). The relationship between humidity and two species (*H. flavus* and *H. italicus*) was non-linear, as the probability of presence quickly decreased when humidity was <80% (Fig. S1). Furthermore, a non-linear relationship between temperature and *H. strinatii* indicated a sharp drop of suitability above 20 °C (Fig. S1). Multiple regression models confirmed the univariate analyses: all species were associated with dark sectors characterized by high humidity and/or low temperature (Table S1).

**Figure 4:**
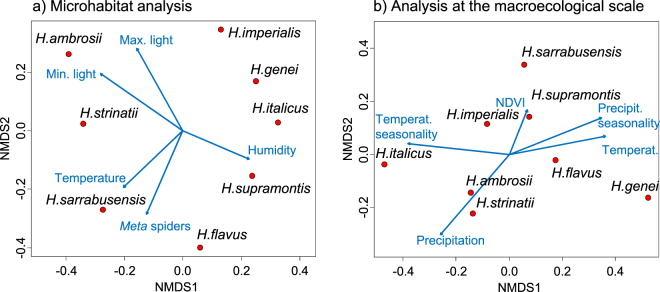
Niche differences among salamander species according to (**a**) microhabitat and (**b**) broad-scale bioclimatic analyses (multidimensional scaling plots). Dots represent the scores of species in the multivariate space; blue arrows are environmental variables added to plots using vector fitting.

**Table 2:** Relationships between the occurrence of eight species of salamanders in underground sectors and microhabitat features.

Species	Humidity			Temperature			Min. Light			Max. Light			Spider presence		
	*B*	*χ* ^2^	*p*	*B*	*χ* ^2^	*p*	*B*	*χ* ^2^	*p*	*B*	*χ* ^2^	*p*	*B*	*χ* ^2^_1_	*p*
*H. ambrosii*	3.5	6.4	**0.012**	−0.14	10.2	**0.001**	−0.6	12.1	**<0.001**	−0.4	15.5	**<0.001**	−0.29	0.2	0.644
*H. flavus*	Q	32.6	**<0.001**	−0.57	12.7	**<0.001**	−76.4	20.8	**<0.001**	−36.6	32.0	**<0.001**	0.20	0.1	0.741
*H. genei*	20.5	16.2	**<0.001**	−0.85	7.7	**0.006**	−1.1	8.7	**0.003**	−0.4	6.4	**0.012**	−0.85	1.7	0.192
*H. imperialis*	7.5	14.5	**<0.001**	−0.39	24.5	**<0.001**	−1.8	18.5	**<0.001**	−0.4	13.0	**<0.001**	0.55	0.3	0.598
*H. italicus*	Q	41.8	**<0.001**	−0.24	19.5	**<0.001**	−3.7	48.2	**<0.001**	−0.7	50.2	**<0.001**	−0.38	1.0	0.317
*H. sarrabusensis*	12.8	4.3	**0.037**	−0.57	4.1	**0.043**	−3.8	4.7	**0.030**	−1.5	6.0	**0.014**	2.84	2.5	0.115
*H. strinatii*	6.0	16.7	**<0.001**	Q	25.3	**<0.001**	−0.8	13.4	**<0.001**	−0.4	15.9	**<0.001**	0.27	0.4	0.527
*H. supramontis*	14.9	27.5	**<0.001**	−0.62	26.7	**<0.001**	−3.7	18.1	**<0.001**	−0.6	10.6	**0.001**	−0.33	0.1	0.705

Results of univariate generalized linear mixed models taking into account imperfect detection. *B*: unstandardized regression coefficients. Q: quadratic relationships (see Fig. S1); all the other models are linear. Significant values are in bold. Degrees of freedom are 1 for linear models, and 2 for quadratic models.

Nevertheless, similarity tests showed significant niche differences for nearly all the species pairs. Niche overlap ranged between 0.165 and 0.799. Niche equivalency was rejected in 21/26 pairwise tests, and remained significant after sequential Bonferroni’s correction in 19/26 tests (Table S2a). The majority of non-significant comparisons involved the species with most restricted range and smallest sample size (*H. sarrabusensis*). According to the microhabitat analyses, *H. ambrosii* and *H. strinatii* were the species most tolerant to light and to dry conditions, *H. sarrabusensis* was the species associated with warmest temperatures, while *H. genei*, *H. italicus* and *H. supramontis* were restricted to the darkest, wettest and coldest sectors (Figs 3 and 4, Fig. S2).

### Bioclimatic analysis

We obtained 597 presence localities, widely covering the range of all the species (5–179 records per species; Table 1, Fig. 2b). Niche overlap measured at the bioclimatic level was generally limited (range: 0.001–0.504), and was lower than the overlap measured at the microhabitat level (paired samples *t-*test for unequal variances: *t*_54_ = −6.1, *p* < 0.0001). Niche equivalency was rejected in 25 out of 26 pairwise tests (Table S2b), and the single non-significant test involved the two species with smallest sample size (*H. sarrabusensis* and *H. supramontis*). According to the bioclimatic analyses, *H. ambrosii* and *H. strinatii* were associated with the coldest and wettest climates, while *H. sarrabusensis*, *H. supramontis*, *H. genei* and *H. flavus* were associated with warm and dry conditions (Figs 4 and S3).

### Microhabitat, bioclimatic niche and phylogenetic relationships

**Figure 5:**
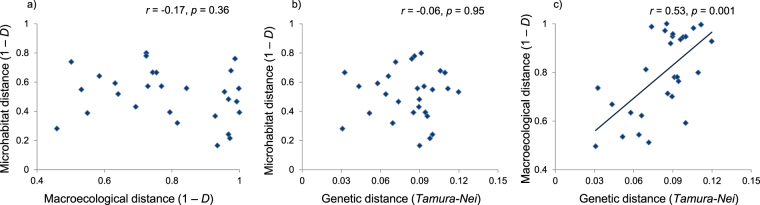
Relationships between microhabitat, bioclimatic, and genetic distances between salamander species. Values on the plots are the results of Mantel’s tests.

The correspondence between microhabitat and bioclimatic niches was weak. For instance, the microhabitat analysis identified *H. strinatii* and *H. ambrosii* among the species with the highest tolerance to dry sectors, while in the bioclimatic analyses they were associated with the wettest climates. Similarly, in the microhabitat analysis *H. genei* was associated with the coldest sectors, while in bioclimatic analyses it was among the species living in the warmest climates (Fig. 4). Overall, we found no relationship between niche dissimilarities calculated using the fine- and the coarse-scale approaches (Mantel’s test: *r* = −0.17, *p* = 0.36, Fig. 5a).

Phylogenetic analyses^32^ showed that the eight study species form a monophyletic group. *H. genei* was the most basal species; two well supported monophyletic groups included (1) *H. flavus*, *H. supramontis*, *H. imperialis* and *H. sarrabusensis* and (2) *H. italicus*, *H. ambrosii* and *H. strinatii*^32^ (Fig. S4).

Microhabitat distances were unrelated to genetic distances (*r* = −0.06, *p* = 0.95, Fig. 4b), while genetically distant species showed the largest bioclimatic distances (*r* = 0.53, *p* = 0.001, Fig. 4c). However, the relationship between bioclimatic distance and evolutionary history was complicated by the fact that species genetically distant also live in distant geographical areas (*r* = 0.47, *p* = 0.013), and bioclimatic distance was positively related to geographical distance between species ranges (*r* = 0.52, *p* = 0.01). Altogether, geographical and genetic distances explained bioclimatic distance well (MRDM: *R*^2^ = 0.39, *p* < 0.003), but disentangling their relative role was difficult. In a commonality analysis, both variables showed a limited unique effect (genetic distance: unique effect = 0.12; geographical distance: unique effect = 0.11), while more explanatory power was shared between these two parameters (Table S3). These results were robust to different approaches to the calculation of niches at both the microhabitat and bioclimatic level, to the incorporation of parameters representing spatial autocorrelation, and to the use of only a subset of localities for analyses (Supplementary Results).

## Discussion

Both microhabitat (i.e. fine-scale) and bioclimatic (i.e. coarse-scale) analyses identified clear niche differences between species. However, the bioclimatic and microhabitat approaches showed dissimilar patterns, as the bioclimatic analyses suggested a close relationship between niche and evolutionary divergence, i.e. a strong phylogenetic signal of niches, while the microhabitat divergence was unrelated to either phylogeny or to the bioclimatic pattern.

Theory clearly acknowledges the multi-scalar nature of niches, and several studies have shown that species distribution is the product of processes acting at both broad and fine scale (reviewed in ref.^33^). An increasing number of studies has tested whether ecological niches retain a signal of phylogenetic history, and many of them have used a bioclimatic approach for niche definition^5^. However, the geographical distribution of organisms is strongly related to their evolutionary history, and recent work suggests that complex interplay between present-day distribution, evolutionary history, and the spatial autocorrelation of bioclimatic variables may complicate the reconstruction of niche evolution^34^. Warren *et al*.^34^ proposed a conceptual framework, in which diversification mostly occurs through allopatric speciation. Sister-species are thus generally allopatric, and only phylogenetically distant species may have overlapping ranges, because they have limited competition. Under this framework, closely related clades may show the strongest apparent niche divergence, even if the opposite may be true (e.g., unrelated species exist in sympatry because of limited competition, i.e. small niche overlap)^35^. Our study shows that a similar interplay between evolutionary history and geography may even determine the opposite pattern. Allopatric speciation was the most likely driver of the differentiation between terrestrial salamander species^36,37^, but strong interspecific competition^38^ and barriers likely cause the absence of sympatry between closely-related species (Fig. 1), while poor dispersal limits their geographical spread. Under these conditions, closely related species often have proximate ranges, and this may cause a pattern with closely related species sharing similar niches (Fig. 5c) just as a by-product of geographical proximity. As a consequence, niche comparisons on the basis of bioclimatic data only can miss the full history: the geography of speciation might be the actual driver of most observed patterns on niche evolution, instead of the inferred ecological processes^34^. The niche comparison method used here^39^ is considered to be able to correct at least in part for the similarity determined by spatial autocorrelation^34^, yet, a clear effect of geographical distance on bioclimatic niche differentiation remained evident. Actually, it was hard to tell whether the niche similarity between closely related species was the result of niche conservatism, or whether it was just the by-product of related species having nearby ranges (Table S3).

The analyses of niches can be improved by the explicit integration of multiple approaches. Measures more closely related to the fundamental niche (e.g. performance, microhabitat selection, tolerance limits, operational conditions), if available, can be used to test the reliability of bioclimatic analyses^40^. For instance, in terrestrial salamanders, the average operational temperature measured at the microhabitat level was unrelated to the average air temperature during the activity season, obtained from global gridded data (Fig. S7), and such discrepancy casts doubts on the reliability of the bioclimatic results alone. On the other hand, the growing availability of spatial datasets and analytical tools allows quickly extracting information that would be much harder to obtain at the microhabitat level, and this has likely helped the fast progress of macroecological studies. Joint availability of broad-scale and fine-grained data is limited^6^, and researchers need to assess the validity of macroecological analyses, even in the absence of information on performance at the small-scale. If the relationship between niche and history abruptly changes when taking into account geography, or if we cannot tease apart their relative role, then Warren’s^34^ hypothesis that we are mistaking geography for biology is a likely explanation. Spatial patterns are inherently linked to ecological processes; thus researchers must utilize approaches that allow explicitly take into account the spatial structure of their data. For instance, the simple effect of geographical distance may be considered as a null-model, over which the phylogenetic history can be compared^41^, even though the spatial effect of past geography, topographical and ecological barriers may be complex, and it is not so easy to explicitly take them into account.

Microhabitat and macroecological analyses certainly characterize non-identical aspects of the niche, still parameters such as thermal preferences have relevant implications on broad scale species distribution^42^, and thus we expected some relationships between them. We suggest that in our study system the microhabitat approach might represent adequately species niches because (*i*) at least for some parameters (e.g. temperature), microhabitat is an excellent proxy of operative eco-physiological conditions of salamanders^26^, which are a major approach to the measurement of fundamental niches^42^; (*ii*) the microhabitat approach is not biased by dispersal limitations or biotic interactions and (*iii*) within each cave, a full range of conditions generally exists, from the harshest to the most suitable, enabling a parallel with habitat preference experiments (Fig. 1). However, it is important to recall that the observed distribution and niche exploitation of species is determined by the joint effect of environmental suitability and dispersal^43,44^. In suboptimal habitats populations can have low fitness, but can be maintained demographically by immigration from nearby source habitats^45,46^. At the macroecological scale, flow of individuals can occur from the centre to the edge of geographical ranges^47^. Similarly, at the microhabitat scale salamanders can be present in suboptimal sectors just because they are nearby sectors with very high abundance, and these issues can complicate the interpretation of niche analyses. Our conclusions are probably robust to these issues, as we obtained identical results both removing observations in sectors with extreme conditions, and removing many distribution records at the boundary of species ranges (Tables S4 and S5). Nevertheless, taking into account variation in abundance across sites can provide a more complete understanding of niche variation in these species, and on the links between environmental suitability at broad scale, and population processes occurring at the local scale^48^.

Niche analyses are increasingly used to answer multiple ecological and evolutionary questions, such as predictions of species’ responses to climate change, analyses of biodiversity drivers and even to analyse local adaptations and identify species. Studies combining distribution data with macroecological predictors can be extremely effective, and some of them have been able to analyse thousands of species at the continental or even global scale. Such broad scale analyses are based on the assumption that grid-cell average climatic conditions provide a good prediction of the probability of species persistence in a site^13^ but, in many cases, this assumption is untested. A few studies have evaluated whether species fitness can be actually predicted by broad-scale analyses (e.g.)^19,49^, and found mixed results. For instance, Searcy and Shaffer^19^ tested whether climatic variables important in broad-scale species distribution models are also related to salamander recruitment, and observed some match between the two approaches. However, the strength of the match was strongly dependent on metrics and methods used to develop the distribution models, and different approaches yielded non-identical predictions of species responses to climate change^19^.

Differences between micro- and macrohabitat approaches might be particularly relevant for animals living in complex landscapes and specific microhabitats (e.g. underground, in freshwater habitats, within plants…) where conditions can be very different from the commonly used measures of climate, such as mean air temperature^10,14,16,30,31^. Actually, such organisms include many amphibians, insects^12^ and likely other terrestrial invertebrates. These taxa are not those most studied in macroecology^6^, but comprise the majority of terrestrial animals, thus the discrepancy between microhabitat and bioclimatic analyses may be present for many organisms. It should also be noted that there are systems in which this pattern was not observed, as some studies on surface-living salamanders found concordance between fine-scale (microclimate, body temperature) and bioclimatic data^20,50^.

It might also be argued that animals associated with underground environments are special cases, if they shelter in microhabitats that are independent from macrohabitat conditions. However, underground temperature and water availability are tightly linked to outdoor temperature and precipitation^27–29^. In the study system, the temperature measured inside caves sectors is strongly related to the surface average annual temperature values (such as the ones used in macroecological analyses) (Fig. S8). The similarity between air temperature inside caves and the average annual outdoor temperature is strikingly high in sectors far from the surface (Fig. S8b), except than in a few outlier caves, which probably have particular air circulation^51^. Underground environments receive a much lower interest in the macroecological/biogeographical literature than more visible aboveground habitats, but host a major portion of Earth biodiversity^52^. Additional analyses are required to assess how frequent are the differences between micro and macrohabitat patterns of niche similarity, and whether our results can apply to different systems.

Macroecology has allowed us to move from reductionist, small scale ecology to a much broader approach with great potential for generalization, which can provide key responses to the global biodiversity crisis^53,54^. Nevertheless, when laying the foundations of macroecology, Brown^53^ described himself as an oddball that continues combining reductionist and holistic approaches. The microhabitat and bioclimatic approaches provide insights about different aspects of species niches, and should be integrated for a more complete understanding of niche variation. The integration of multiple approaches certainly requires more time and investments, but the urgency to obtain answers should not preclude the need of robust, biologically sound data^55^. The integration of studies at multiple scales allows to take into account a broader spectrum of processes influencing populations, thus providing more accurate inference on niche evolution^19^. A better combination between bioclimatic and fine-grained data^56^, and also considering additional niche components such as diet and other biotic interactions, may be a key to obtain robust generalizations that can help us to address the consequences of global changes.

## Methods

### Study system

In summer, underground environments show a continuous microclimatic gradient: the superficial sectors have conditions similar to the outdoor ones (light, high temperature, low humidity). However, far from the surface the microhabitat becomes wetter, colder and dark (Fig. 1). Salamanders move underground because they must reach the sectors where conditions are within the tolerance limits of the species^12^ but, as food is more abundant in superficial sectors^28,57,58^, they are restricted to a few tens of meters from the surface. Generally, the realized niche does not correspond to the fundamental niche because of dispersal limitations and biotic interactions^43^. These issues exist for all the environments^33^ but, within this system, they are alleviated because the full environmental gradient exist within a few meters, well within the dispersal ability of individuals, and because of the lack of predators and competitors within these environments (*Hydromantes* species are allopatric, Fig. 2, no other terrestrial salamanders are present, and they are apex predators in these environments)^59^. Movements are limited and home ranges small (6–22 m^2^)^25^, therefore observations are unlikely to represent transient individuals. The study system thus can be viewed as a natural habitat selection experiment, in which individuals are exposed to continuous environmental gradients, within which they select the favourable conditions (i.e., the conditions within their fundamental niche). Furthermore, previous studies showed that the microhabitat conditions selected by salamanders are consistent through the year, and niche estimates from summer surveys are generally similar to estimates for the other seasons^28^. Summer is the period in which salamander detection is easiest, thus analyses performed on summer observation allow an appropriate characterization of species niche. Finally, terrestrial salamanders are generally at equilibrium with their environment for temperature and water and, in the field, the average temperature difference between air and body temperature is <0.5 °C^26,60^. Thus, air conditions are an excellent proxy of operative conditions of individuals^12,26^.

### Ethics statement

Samples were collected in accordance with regulations for the protection of terrestrial wild animals (authorization by the Italian Ministry of the Environment, prot. 0040002).

### Surveys and data collection

To measure species distribution and habitat at fine spatial scale (microhabitat) we surveyed caves in Mediterranean Italy and France, widely covering the range of all European *Hydromantes* species (Fig. S1a). We excluded caves from the narrow hybrid zone between *H. ambrosii* and *H. italicus*^61^. Surveys were performed in early summer (June–July 2011–2014), when the conditions outside the cave are unfavourable and underground detection is highest^28^. All surveys were performed during the central hours of sunny and dry days. Each cave was subdivided in 3-m longitudinal intervals (hereafter: sectors); the size of sectors approximately corresponds to home ranges size^25,57^, covering the whole cave or until the first empty sector after the last salamander. Overall, we surveyed 278 caves and 1251 cave sectors. In each sector we used visual encounter surveys to detect the presence of active salamanders, and measured four abiotic variables known to influence salamander distribution: air temperature (°C; accuracy: 0.1 °C) and relative humidity (%; accuracy: 0.1%) were recorded with a EM882 multi-function device, waiting until the measurement was stable (variation <0.1 °C or <0.1% for >60 seconds). Minimum and maximum incident light (illuminance, measured in lux, accuracy 0.01 lux) were recorded using the EM882 by performing at least 10 measures of illuminance in the portions of the sector receiving more and less light, respectively. Furthermore, as a biotic parameter, we counted the number of adult large *Meta* spiders (*M. menardi* or *M. bourneti*). These spiders are the major predators of arthropods in the study caves, and have been proposed as indicators of prey availability for salamanders^57,62^.

To analyse the bioclimatic niche, we obtained distribution records covering the whole range of all the *Hydromantes* species from the present study and from the literature^25,37,61,63–66^. We only considered localities with accuracy of 1-km or better. To match the number of microhabitat predictors, we considered five bioclimatic parameters: mean temperature and summed precipitation during the period in which salamanders are active outside the cave (from September to May), temperature seasonality, precipitation seasonality, and normalized difference vegetation index (NDVI). Climatic variables were extracted at the 30 arc-second resolution from Worldclim^67^, while NDVI was extracted from the ESA Land Cover CCI (mean NDVI over the 1999–2012 period; http://maps.elie.ucl.ac.be/CCI/viewer/download.php). Tolerance to these parameters is assumed to directly influence animals, particularly during the periods in which they perform outdoor activity. To assess the robustness of our conclusions to the selection of parameters, we also repeated analyses using annual climatic features.

### Microhabitat preferences of species

We used generalized linear mixed models (GLMMs) with binomial error to assess the within-cave relationships between each species and the features of cave sectors. In GLMMs, cave identity was included as random effect, salamander presence as dependent, and the five microhabitat variables were the predictors. First, for each species we built the univariate models relating salamander presence to the five microhabitat variables. We tested both linear and quadratic relationships; quadratic terms were retained if they significantly improved fit. We then used the Akaike’s Information Criterion (AIC) to build the minimum adequate models, best describing the occurrence pattern of each species on the basis of multiple predictors^68^. We built models considering all possible combinations of microhabitat variables, and ranked them using AIC. Some microhabitat variables were strongly correlated: minimum illuminance was related to maximum illuminance, while temperature data were negatively correlated with humidity (in the datasets of most species, |*r*| > 0.7). Models including highly correlated variables were excluded from the candidate models. The lowest-AIC model, i.e. the one explaining more variation with fewer predictors, was considered as the minimum adequate model for each species^68^.

A species is certainly present where it is detected, while non-detection may represent either real absences or failure of detecting the present species; not taking into account misdetection can influence regression results^69^. Previous analyses on a subset of species showed that, with our sampling protocol, detection probability is high but imperfect (approx. 0.75 per visit)^28,70^. Therefore, in our models we weighted absences with a weight of 0.75 following^71^. We calculated significance of variables using likelihood-ratio tests. For all species the residual deviance was similar or lower than the residual degrees of freedom (variance inflation factor of best-AIC models always ≤1.06), therefore overdispersion was not an issue. Before running analyses, illuminance was log-transformed, while humidity % was transformed using square-root-arcsine to improve normality and reduce skewness.

### Niche overlap and equivalency among species

We used an approach based on Principal Component Analyses of environmental variables (PCA-env) to perform multivariate comparisons of niche overlap between pairs of species following^39^. PCA-env measures niche overlap between pairs of species or populations on the basis of occurrence and environmental data, is among the most reliable techniques for niche comparisons, and shows better performance than approaches based on species distribution modelling^39^. PCA-env uses a kernel density function to compute the density of occurrences in the multivariate PCA space, in order to take potential bias into account that stems from unequal sampling effort. We calculated niche overlap and equivalency using the Schoener’s *D* metric^72^. Schoener’s *D* ranges between 0 (lack of overlap) and 1 (complete overlap), and is among the most widespread metrics of niche overlap in ecological, evolutionary and biogeographical studies (e.g.^72,73^). For the niche comparison of a species pair, PCA-env performs a non-phylogenetic principal component analysis (PCA) on the environmental spaces available to the two species^39^. In the micro-habitat analysis, the “available space” of each species corresponded to the sectors of all the surveyed caves within the range of that species. In the bioclimatic analysis, the available space corresponded to the grid cells within 150 km from known presence points. This distance is three times the largest gap within a species range, thus likely includes all the areas potentially available to species dispersal (see^44^). Preliminary analyses using different distance buffers yielded highly consistent results (see also^74^).

Species distribution data and bioclimatic variables often show strong spatial autocorrelation, and this can influence the outcome of ecological analyses, but no formal approaches are currently available to incorporate autocorrelation into PCA-env. To assess the robustness of PCA-env to spatial autocorrelation, we repeated the bioclimatic analysis including an additional predictor representing spatial autocorrelation. For each species, we first built a spatial generalized additive model (GAM) with binomial error, using species presence/absence as dependent variable, and incorporating geographic coordinates of sites as tensor product smooth terms, using thin plate regression splines^75^. We then used the spatial predictions of GAMs as an additional covariate in PCA-env. Even though the incorporation of spatial predictions as covariates is not a perfect approach to deal with autocorrelation, simulations showed that this implementation of GAMs helps to correctly estimate relationships in spatially structured datasets with relatively good performance^75^. For both the microhabitat and bioclimatic analyses, significance of niche differences between species was assessed using the niche equivalency tests through 1999 permutations.

### Relationships between microhabitat, bioclimatic niche and evolutionary history

Genetic distance between species pairs was calculated on the basis of three mitochondrial (12S, 16S and cyt-*b*) and two nuclear (RAG-1 and BNDF) genes, amplified by van der Meijden *et al*.^32^. We considered the 49 individuals for which data from all five genes were available (2–15 individuals per species). The concatenated genetic dataset contained 3494 base pairs^32^. The Tamura-Nei distance was calculated for each species pair, using the between group mean distance function in Mega 6. To calculate the geographical distances among species, we generated the polygon of the range of each species on the basis of presence records using α-hulls^76^, and then calculated the Euclidean distances between the centroids of the ranges.

Microhabitat and bioclimatic niche distances between species were calculated as 1 - Shoener’s *D*. We then evaluated the relationships between microhabitat, bioclimatic and genetic distances. First, we used non-metric multidimensional scaling (NMDS) for the graphical representation of niche distances among species^77^. For the graphical representation of among-species differences in habitat relationships, we calculated the mean values of environmental variables in the presence localities, and then fitted them to the NMDS space using vector fitting^78^. Vector fitting returned essentially the same niche differences between species obtained with PCA-env (Figs S2, S3), with the advantage of synthetically illustrating the relationships between all the species pairs in one single plot. Relationships between niche dissimilarity at micro- and macro-ecological level and genetic differentiation were analysed with Mantel’s test for ranked data (bivariate analyses) or with rank multiple regressions on distance matrices (MRDM; multivariate analyses)^77^, using 9999 permutations to assess significance. Previous studies have shown that the Mantel test and other metrics of phylogenetic signal (e.g. Abouheif index, Bolmberg’s K) are closely related to each other because they are all based on a cross-product statistic, and the Mantel test is thus appropriate to assess phylogenetic signal for dissimilarity matrices^79–81^. After MRDM, we used commonality analysis to assess the unique and common contribution of intercorrelated independent variables^82^. Statistical analyses were run using the packages lme4, MuMIn, raster, vegan, ecodist and hyat in R 3.1 (www.r-project.org).

### Data availability

Raw data are available as Data table S1. To avoid illegal poaching on protected species, we degraded the quality of distribution records. The reported coordinates have a randon error of up to 3 km, compared to the true ones. The correct coordinates were used for analyses.

## Electronic supplementary material

Supporting Information

Supplementary data
