# Supplementary material for: Differences between microhabitat and broad-scale patterns of niche evolution in terrestrial salamanders
Source: Sci Rep. 2018 Jul 12;8:10575. doi: 10.1038/s41598-018-28796-x (PMC6043550; doi:10.1038/s41598-018-28796-x)

Supplementary material

Differences between microhabitat and broad-scale patterns of niche evolution in terrestrial salamanders

Gentile Francesco Ficetola, Enrico Lunghi, Claudia Canedoli, Emilio Padoa-Schioppa, Roberta Pennati & Raoul Manenti

SUPPLEMENTARY RESULTS

Niche analyses repeated measuring niches using different approaches

Microhabitat niche measured using the tolerance limit approach

Species distribution is often defined by tolerance limits for the fundamental niche. For instance, the northern limit of species may be defined by the tolerance limit to low temperatures e.g. [1,2]. The approach to microhabitat niche in the main text compared the conditions at all the presence points where individuals were observed. We repeated the analyses by using the limits instead. For all study species, within cave distribution was determined by temperature, humidity and light, being positively related to humidity and negatively related to light and temperature within the observed range of these variables (Fig. 3, Table 2). We therefore calculated the tolerance limit of each species as the value of each variable below which there are 95% of salamander records (for temperature and light), or the value above which there are 95% of records (humidity). For instance, for *H. flavus* the tolerance limit for temperature and humidity were 18.1°C and 88.4%, respectively, because

95% of sectors with salamanders showed temperature ≤ 18.1°C and humidity ≥ 84.4%. The between-species differences in tolerance limits were then calculated as the pairwise Euclidean distances. Spiders were not considered in this analysis, as we never found clear relationships between spiders and salamanders (Table 2).

Microhabitat distance measured using the tolerance limit approach (Table S5) was positively correlated to microhabitat distances on the basis of Shoener's *D* (Mantel's test: r = 0.45, p = 0.01), while it was unrelated to genetic distance (r = 0.001, p = 0.997) and was not positively related to bioclimatic distance (r = -0.30, p = 0.26), confirming the results presented in the main text.

Bioclimatic niche measured using annual climatic features

We repeated the bioclimatic analysis using mean temperature and summed annual precipitation, instead than temperature and precipitation measured in the activity period (autumn, winter and spring). Niche overlap using annual features (Table S4) was nearly identical to the overlap obtained considering climate during the activity period (Mantel's test: r = 0.98, P = 0.0001; compare Fig. 3 with Fig. S3). Bioclimatic distance measured using annual climate was unrelated to microhabitat distance (r = -0.2, p = 0.28), while it remained positively related to both genetic (r = 0.49, p = 0.004) and geographical distance (r = 0.56, p = 0.004), confirming the results in the main text.

Bioclimatic niche measured incorporating spatial autocorrelation

Spatial autocorrelation might affect the results of PCA-env, therefore we repeated the bioclimatic analysis also including the prediction of a spatial generalized additive model as an additional variable. Niche overlap measured using this additional variable (Table S4) was strongly correlated to the overlap measured using standard PCA-env (Mantel's test: $r = 0.75$, $p = 0.0006$). Also in this analysis, bioclimatic distance was unrelated to microhabitat distance ($r = 0.003$, $p = 0.99$), while it remained positively related to both genetic ($r = 0.60$, $p = 0.002$) and geographical distance ($r = 0.75$, $p = 0.0002$), confirming the results in the main text.

Bioclimatic analyses performed considering only presence points nearby to localities used for microclimatic analyses.

The distribution of surveyed localities (Fig. 2a) was not identical to the distribution of points available from bioclimatic analyses (Fig. 2b). Therefore, we repeated the bioclimatic analysis using only localities that are nearby (within 15 km) points used in microhabitat analyses. This resulted in a much smaller sample size ($N = 251$ localities; the overall sample size available for bioclimatic analyses was $N = 591$). Furthermore, with this approach sample size was much more homogeneous among species. For instance, for *H. strinatii* 177 localities were available for bioclimatic analyses (Table 1), while only 64 localities were available in this reduced-dataset. In the complete dataset (Table 1), the standard deviation of the sample size available across species was 58.4, while in the reduced-dataset the standard deviation was 19.2.

Niche overlap measured by using only these localities (Table S5) was strongly correlated to the overlap measured using standard PCA-env (Mantel's test: $r = 0.90$, $p < 0.0001$). Also in

this analysis, bioclimatic distance was unrelated to microhabitat distance (r = -0.15, p = 0.415), while it remained positively related genetic distance (r = 0.49, p = 0.003), confirming the results in the main text. The analysis of the reduced-dataset also yielded a highly consistent multi-dimensional scaling (correlation between rotated NMDS plots = 0.85, p = 0.0001; Fig. S6).

Supplementary references

1 Whittaker, R. J., Willis, K. J. & Field, R. Scale and species richness: towards a general, hierarchical theory of species diversity. *J. Biogeogr.* **28**, 453-470, doi:10.1046/j.1365-2699.2001.00563.x (2001).

2 Hutchinson, G. E. Population studies: animal ecology and demography. Concluding remarks. *Cold Spring Harbor Symposia on Quantitative Biology* **22**, 415-427 (1957).

3 Nakagawa, S. & Schielzeth, H. A general and simple method for obtaining R^2 from generalized linear mixed-effects models. *Methods Ecol. Evol.* **4**, 133-142, doi:10.1111/j.2041-210x.2012.00261.x (2013).

4 Richards, S. A., Whittingham, M. J. & Stephens, P. A. Model selection and model averaging in behavioural ecology: the utility of the IT-AIC framework. *Behav. Ecol. Sociobiol.* **65**, 77-89 (2011).

5 Adams, D. C. & Nistri, A. Ontogenetic convergence and evolution of foot morphology in European cave salamanders (Family: Plethodontidae). *BMC Evol. Biol.* **10**, 10, doi:10.1186/1471-2148-10-216 (2010).

Table S1. Relationships between the occurrence of nine species of salamanders in underground sectors and microhabitat features: best-AIC generalized linear mixed models taking into account imperfect detection. R^2_C: conditional R^2 (ref. [3]); B: unstandardized regression coefficients. For four species, some uncertainty in model selection occurred. Specifically, for *H. genei* a model without light showed Δ-AIC = 0.45; for *H. sarrabusensis* a model without max. light but with min. light showed Δ-AIC = 1.33; for *H. strinatii* a model without max. light and with min. light showed Δ-AIC = 1.43. For *H. supramontis*, a model without temperature, but including a positive relationships with humidity, showed Δ-AIC = 0.34. For the remaining species, no candidate models showed Δ-AIC < 2, after the removal from the candidate set of those models that were more complicated versions of any model with a lower AIC value [4].

	Best AIC models			
	R^2_C	B	χ^2_1	p
H. ambrosii	0.315			
Max light		-0.44	15.1	<0.001
Temperature		-0.13	9.8	0.002
H. flavus	0.99			
Max light		-29.93	5.3	0.020
Humidity		169.73	5.0	0.025
Humidity (Q)		-61.65	4.4	0.035
H. genei	0.73			
Min. light		-0.63	2.5	0.111
Humidity		17.51	10.1	0.002
H. imperialis	0.39			
Min. light		-1.32	8.6	0.003
Temperature		-0.31	14.6	<0.001
H. italicus	0.57			
Max. light		-0.62	29.1	<0.001
Humidity		8.92	17.8	<0.001
H. sarrabusensis	0.70			
Max. light		-1.45	6.0	0.014
H. strinatii	0.44			
Max. light		-0.39	9.8	0.002
Temperature		1.24	10.6	0.001
Temperature (Q)		-0.04	13.5	<0.001
H. supramontis	0.80			
Min. light		-1.53	3.7	0.055
Temperature		-0.50	12.3	<0.001

Table S2. Niche overlap between terrestrial salamanders, measured at both the microhabitat and the broad scale (bioclimatic) levels. In bold, significance values that remained significant after sequential Bonferroni's correction.

a) Niche overlap at the microhabitat-level

	H. ambrosii	*H. flavus*	*H. genei*	*H. imperialis*	*H. italicus*	*H.sarrabusensis*	*H. strinatii*
				Schoener's *D*			
H. flavus	0.201						
H. genei	0.24	0.443					
H. imperialis	0.451	0.408	0.633				
H. italicus	0.334	0.518	0.608	0.607			
H. sarrabusensis	0.467	0.681	0.322	0.262	0.444		
H. strinatii	0.719	0.221	0.534	0.429	0.429	0.759	
H. supramontis	0.335	0.613	0.835	0.482	0.785	0.359	0.57
				Significance of niche equivalency tests			
H. flavus	**<0.001**						
H. genei	**<0.001**	**<0.001**					
H. imperialis	**<0.001**	**<0.001**	**<0.001**				
H. italicus	**<0.001**	**<0.001**	**<0.001**	**<0.001**			
H. sarrabusensis	0.465	**<0.001**	**<0.001**	0.017	**<0.001**		
H. strinatii	**<0.001**	**<0.001**	**<0.001**	**<0.001**	**<0.001**	0.907	
H. supramontis	0.023	0.554	0.099	**<0.001**	**<0.001**	0.081	**<0.001**

b) Niche overlap at the broad-scale

	H. ambrosii	*H. flavus*	*H. genei*	*H. imperialis*	*H. italicus*	*H.sarrabusensis*	*H. strinatii*
				Shoener's *D*			
H. flavus	0.220						
H. genei	0.028	0.081					
H. imperialis	0.408	0.366	0.065				
H. italicus	0.264	0.052	0.001	0.236			
H. sarrabusensis	0.072	0.188	0.018	0.488	0.004		
H. strinatii	0.504	0.287	0.012	0.219	0.331	0.053	
H. supramontis	0.201	0.465	0.042	0.457	0.055	0.378	0.299
				Significance of niche equivalency tests			
H. flavus	**<0.001**						
H. genei	**<0.001**	**<0.001**					
H. imperialis	**<0.001**	**<0.001**	**<0.001**				
H. italicus	**<0.001**	**<0.001**	**<0.001**	**<0.001**			
H. sarrabusensis	**<0.001**	**<0.001**	**<0.001**	**0.015**	**<0.001**		
H. strinatii	**<0.001**	**<0.001**	**<0.001**	**<0.001**	**<0.001**	**<0.001**	
H. supramontis	**<0.001**	**0.002**	**<0.001**	**<0.001**	**<0.001**	0.086	**<0.001**

Table S3. Results of commonality analysis evaluating the unique, common and total contribution of genetic and geographical distance on the bioclimatic differentiation of niches.

Independent variables	Contribution		
	Unique	Common	Total
Genetic distance	0.116	0.166	0.282
Geographical distance	0.105	0.166	0.272

Table S4. Alternative approaches to the measure of niche overlap between salamander species. a): niche distances at the microhabitat-level: Euclidean distances calculated using species tolerance limits; b) niche overlap at the broad scale (bioclimatic), calculated using annual values of temperature of precipitation; c) niche overlap at the bioclimatic scale, calculated also including a term representing spatial autocorrelation. In bold, significant values after sequential Bonferroni's correction. In a), no significance values can be calculated.

a) Niche distances at the microhabitat-level: Euclidean distances calculated using species tolerance limits

	H. ambrosii	*H. flavus*	*H. genei*	*H. imperialis*	*H. italicus*	*H.sarrabusensis*	*H. strinatii*
H. flavus	29.16						
H. genei	26.07	4.30					
H. imperialis	16.69	15.46	11.72				
H. italicus	26.12	4.01	2.88	11.72			
H. sarrabusensis	29.53	3.89	3.52	14.71	4.90		
H. strinatii	12.21	17.55	14.57	8.93	14.92	18.02	
H. supramontis	23.85	7.65	5.41	8.81	3.67	7.84	13.51

b) Niche overlap at the broad-scale, calculated using annual values of temperature of precipitation

				Shoener's *D*			
	H. ambrosii	*H. flavus*	*H. genei*	*H. imperialis*	*H. italicus*	*H.sarrabusensis*	*H. strinatii*
H. flavus	0.276						
H. genei	0.013	0.157					
H. imperialis	0.469	0.369	0.072				
H. italicus	0.257	0.032	0.001	0.206			
H. sarrabusensis	0.044	0.184	0.025	0.498	0.002		
H. strinatii	0.541	0.276	0.008	0.231	0.271	0.032	
H. supramontis	0.246	0.451	0.065	0.359	0.028	0.415	0.307
				Significance of niche equivalency tests			
H. flavus	**<0.001**						
H. genei	**<0.001**	**<0.001**					
H. imperialis	**<0.001**	**<0.001**	**<0.001**				
H. italicus	**<0.001**	**<0.001**	**<0.001**	**<0.001**			
H. sarrabusensis	**<0.001**	**<0.001**	**<0.001**	**0.017**	**<0.001**		
H. strinatii	**<0.001**	**<0.001**	**<0.001**	**<0.001**	**<0.001**	**<0.001**	
H. supramontis	**<0.001**	**<0.001**	**<0.001**	**<0.001**	**<0.001**	0.113	**<0.001**

c) Niche overlap at the broad-scale, integrating an additional term representing spatial autocorrelation

				Shoener's *D*			
	H. ambrosii	*H. flavus*	*H. genei*	*H. imperialis*	*H. italicus*	*H.sarrabusensis*	*H. strinatii*
H. flavus	0.238						
H. genei	0.069	0.506					
H. imperialis	0.249	0.426	0.235				
H. italicus	0.387	0.217	0.000	0.205			
H. sarrabusensis	0.008	0.247	0.070	0.452	0.009		
H. strinatii	0.349	0.240	0.170	0.162	0.243	0.027	
H. supramontis	0.189	0.468	0.443	0.492	0.174	0.302	0.249
H. flavus	**<0.001**						
H. genei	**<0.001**	**<0.001**					
H. imperialis	**<0.001**	**<0.001**	**<0.001**				
H. italicus	**<0.001**	**<0.001**	**<0.001**	**<0.001**			
H. sarrabusensis	**<0.001**	**<0.001**	**<0.001**	**<0.001**	**<0.001**		
H. strinatii	**<0.001**	**<0.001**	**<0.001**	**<0.001**	**<0.001**	**<0.001**	
H. supramontis	**<0.001**	**<0.001**	**<0.001**	**<0.001**	**<0.001**	**0.020**	**<0.001**

Table S5. Alternative approaches to the measure of niche overlap between salamander species. Niche overlap at the broad scale (bioclimatic), calculated using only presence points nearby localities used for microhabitat analyses.

	H. ambrosii	*H. flavus*	*H. genei*	*H. imperialis*	*H. italicus*	*H. sarrabusensis*	*H. strinatii*
H. flavus	0.184						
H. genei	0.015	0.060					
H. imperialis	0.447	0.344	0.016				
H. italicus	0.281	0.026	0.000	0.286			
H. sarrabusensis	0.030	0.146	0.000	0.504	0.001		
H. strinatii	0.442	0.170	0.027	0.279	0.288	0.083	
H. supramontis	0.194	0.384	0.010	0.319	0.018	0.201	0.202

Supplementary figures

Figure S1. Relationships between microhabitat features and presence of salamanders. The plots show the three relationships that are significantly non-linear (See Table 1).

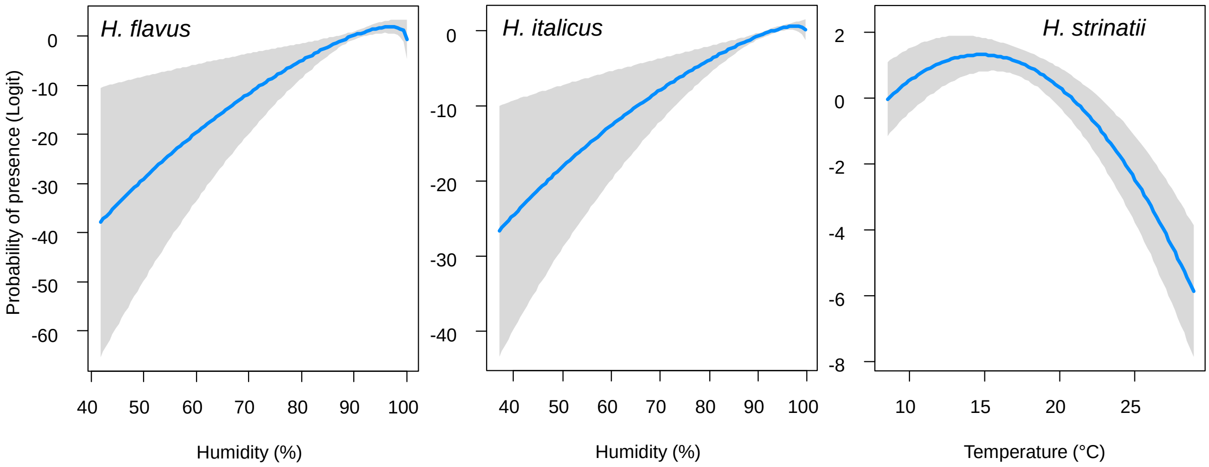

Figure S2. Results of PCA-env, representing the microhabitat niche of the study species in the niche space. The left panels represent the contribution of the climatic variables on the PCA axes, and the percentage of inertia explained by the two axes. Central and right panels represent the niche of the species along the two first PCA axes. Grey shading shows the density of species occurrences by cell. The Shoener's *D* values and the results of similarity plots are reported in Table S3. Abbreviation of variables in the correlation circles are: Temp: temperature; Lux_max / Lux_min: maximum and minumum light illuminance; Humidity: relative air humidity; Meta: presence of *Meta* spiders (either *M. menardi* or *M. bourneti*). For each species pair, PCA-env was re-run on the environmental space available to those two species. Therefore the PCA-env plot of a given species can be different across comparisons.

H. ambrosii – H. flavus

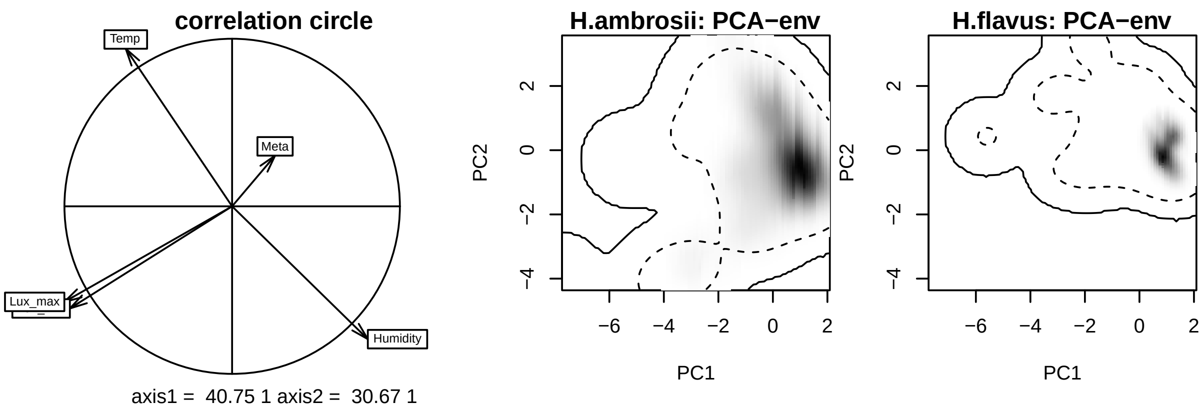

H. ambrosii – H. genei

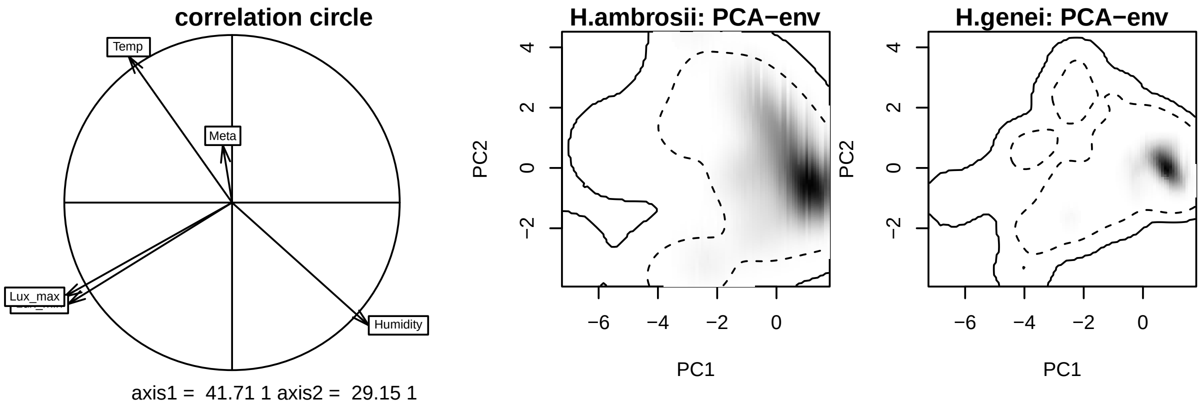

(continues)

H. ambrosii – H. imperialis

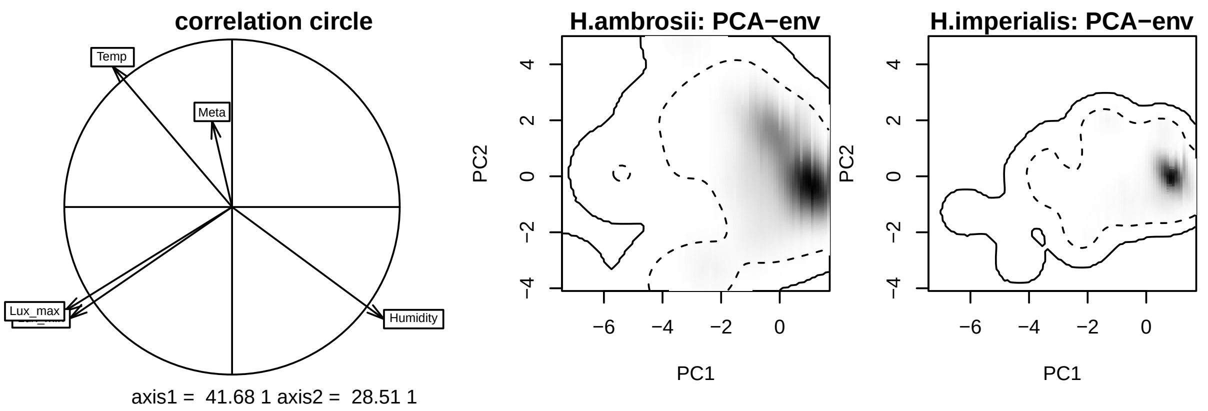

H. ambrosii – H. italicus

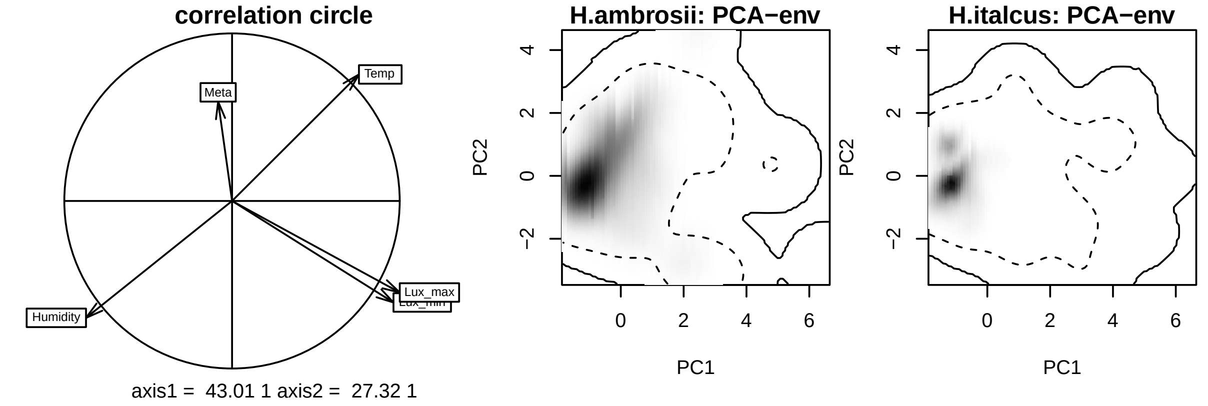

H. ambrosii – H. sarrabusensis

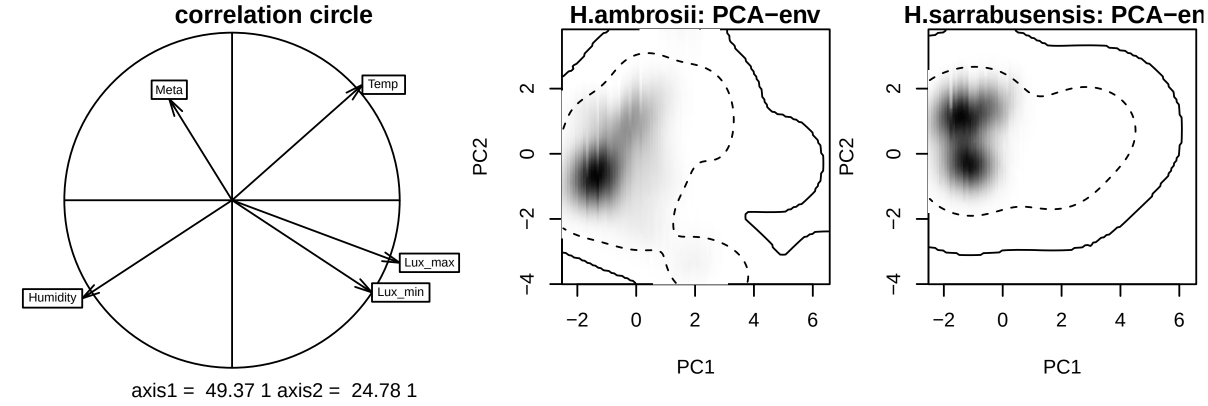

Fig. S2 (continues)

H. ambrosii – H. strinatii

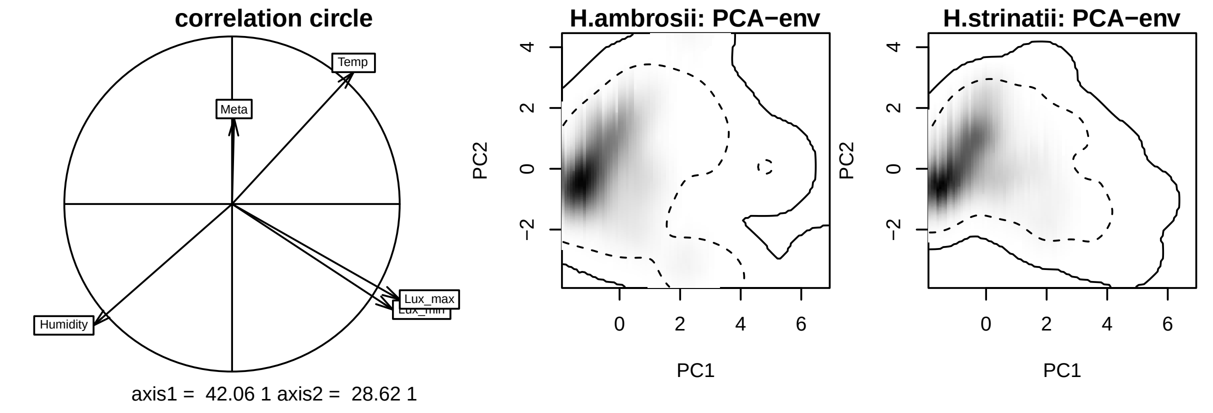

H. ambrosii – H. supramontis

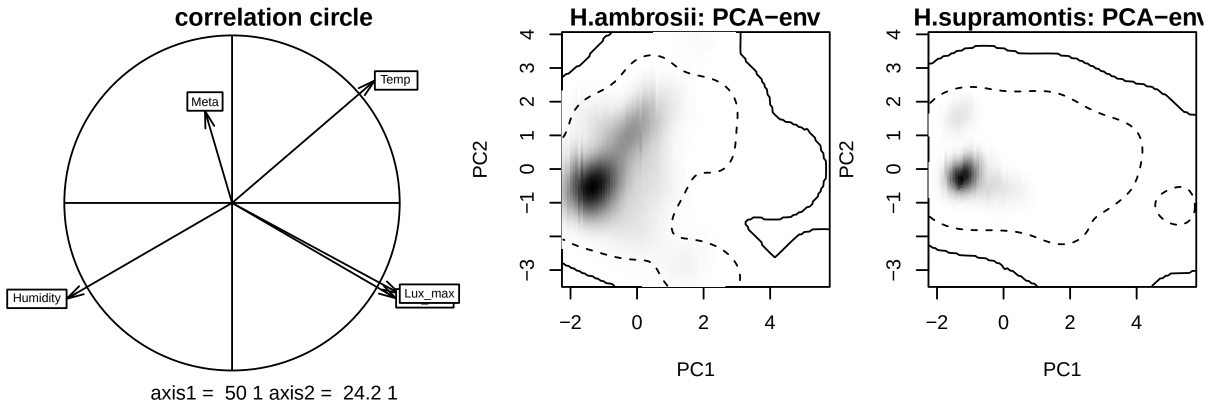

H. flavus – H. genei

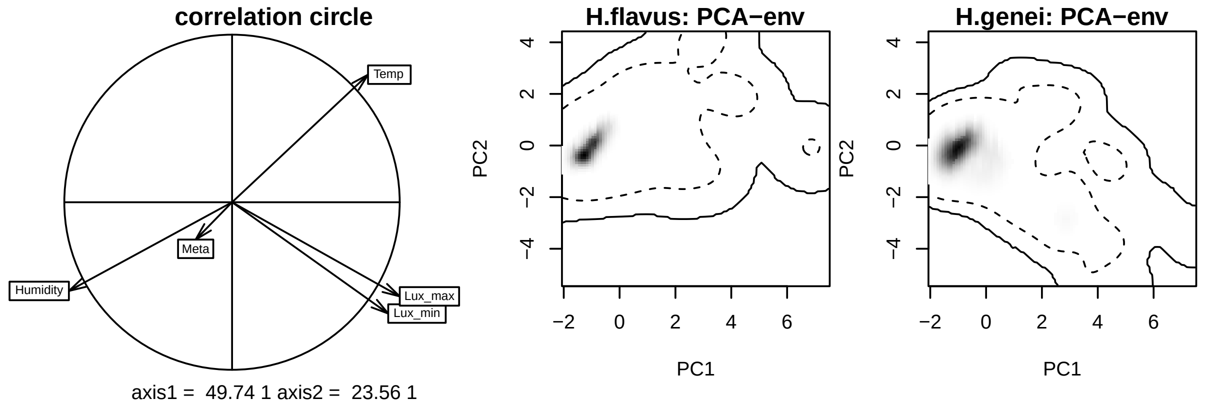

Fig. S2 (continues)

H. flavus – H. imperialis

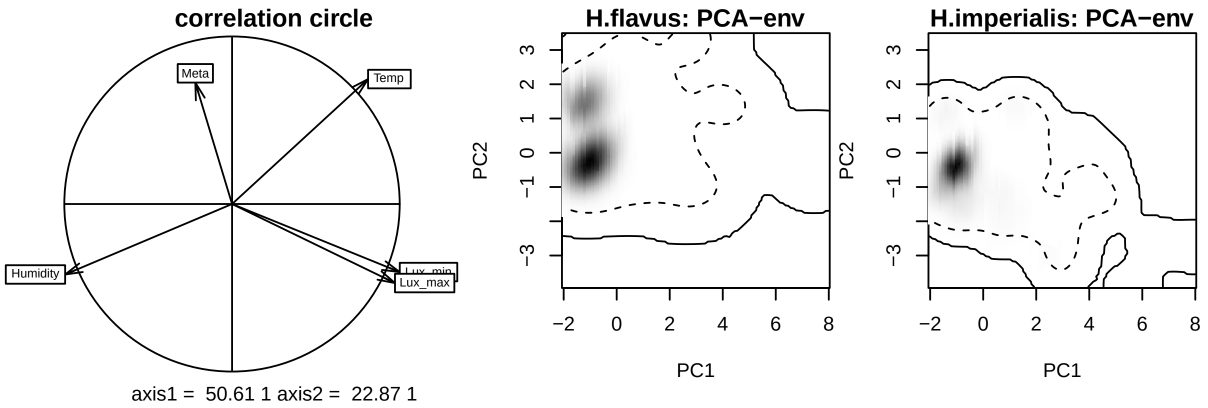

H. flavus – H. italicus

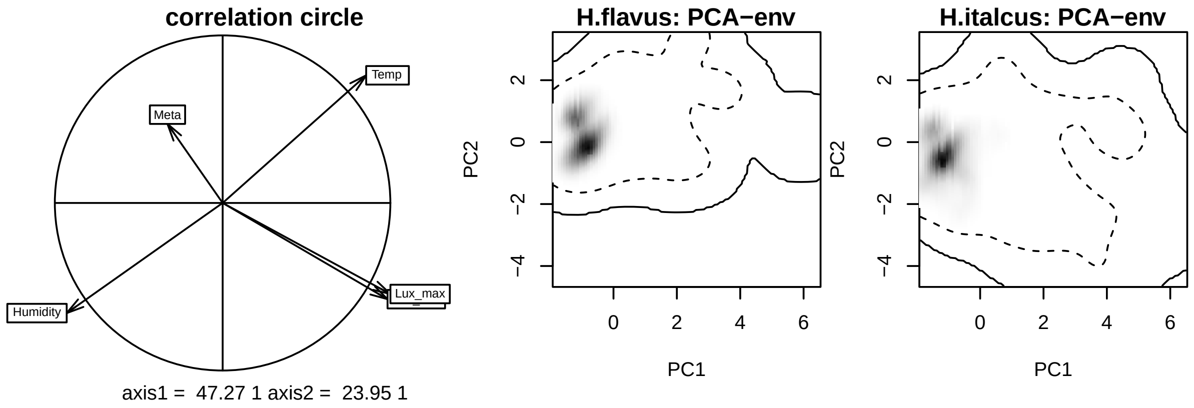

H. flavus – H. sarrabusensis

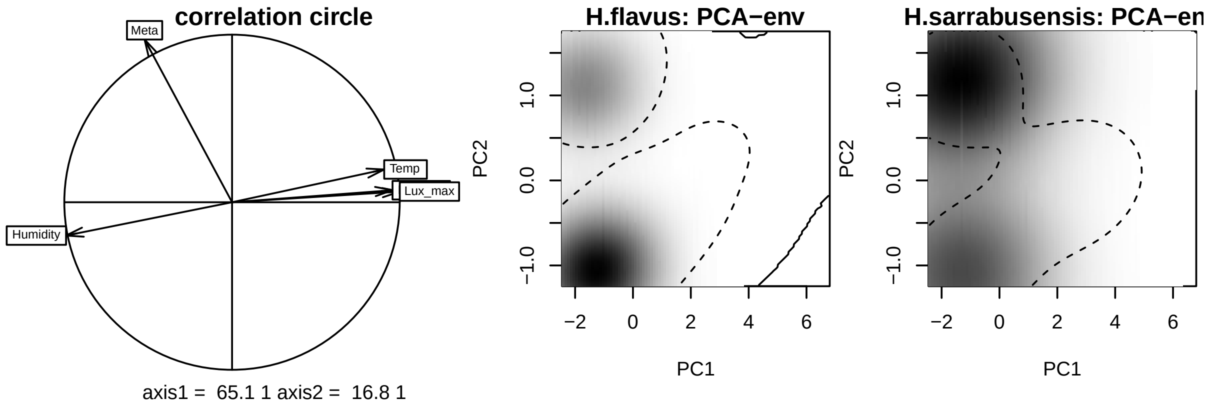

Fig. S2 (continues)

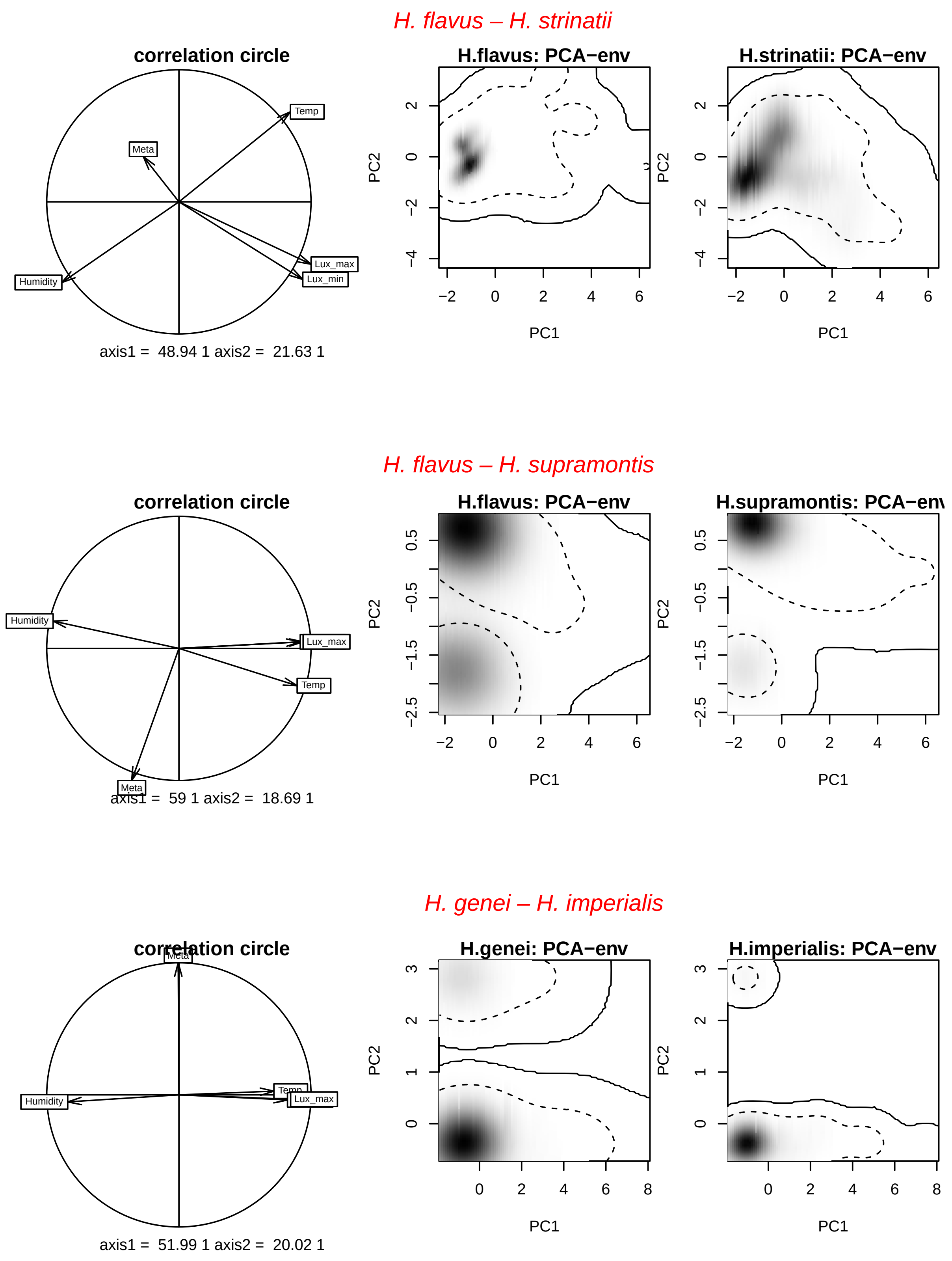

Fig. S2 (continues)

H. genei – H. italicus

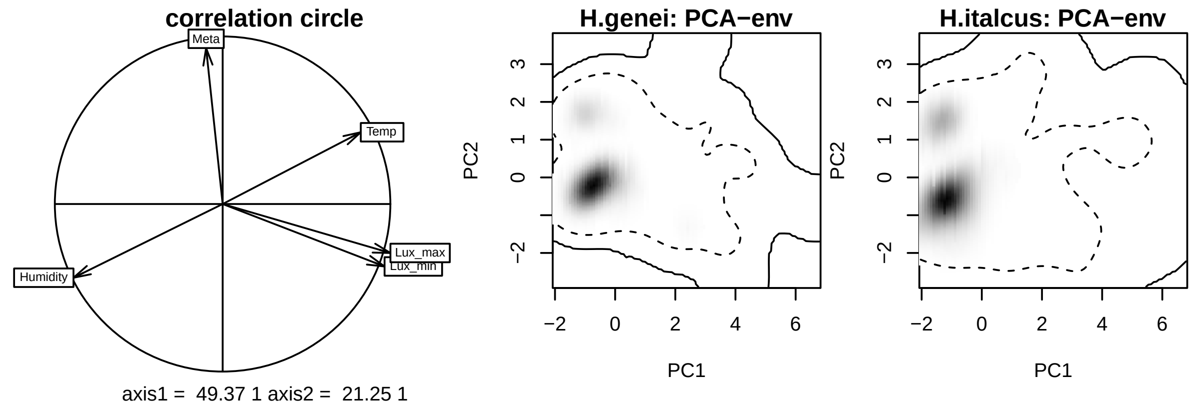

H. genei – H. sarrabusensis

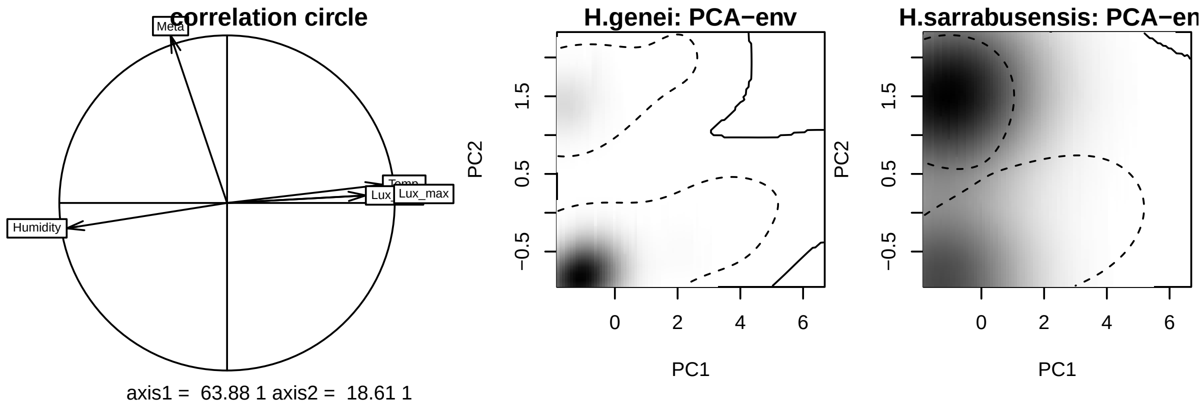

H. genei – H. strinatii

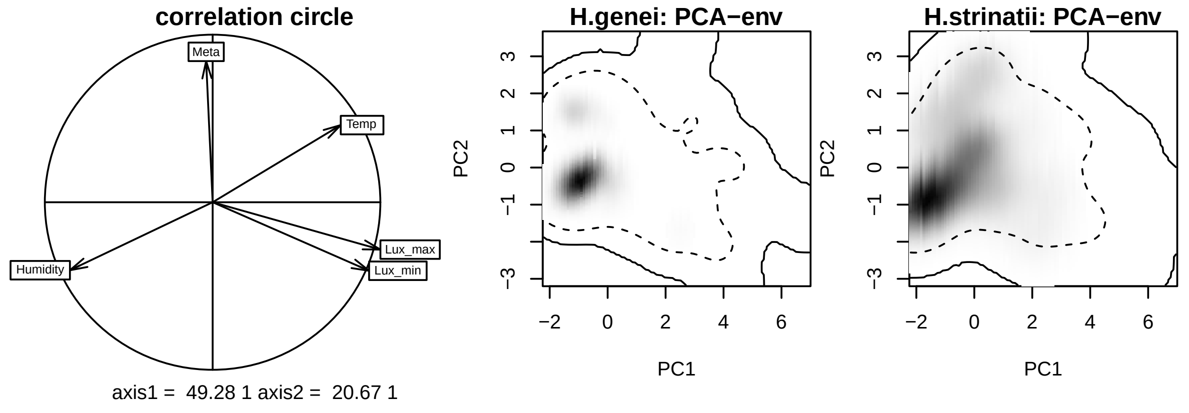

Fig. S2 (continues)

H. genei – H. supramontis

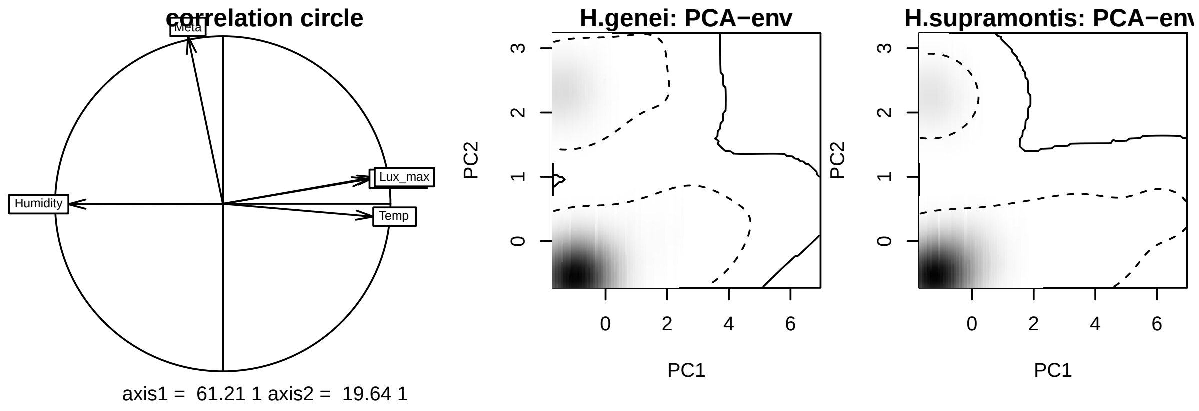

H. imperialis – H. italicus

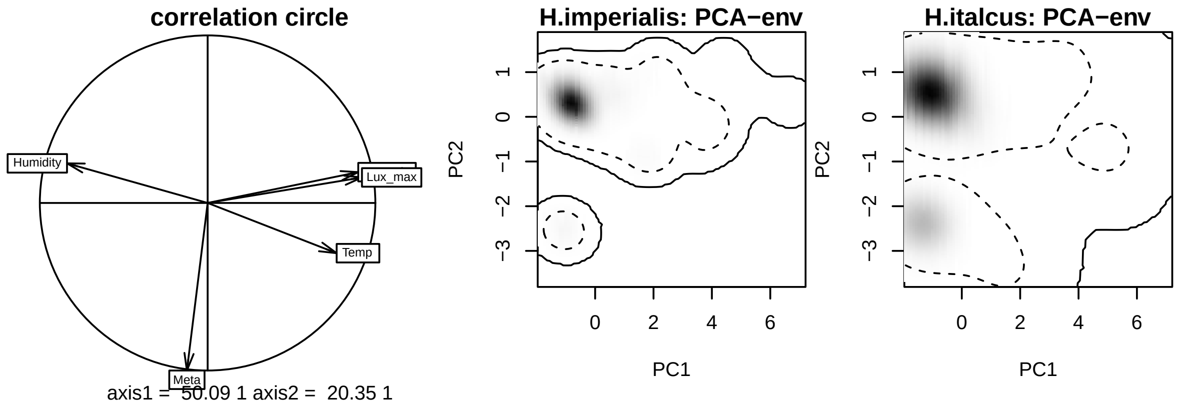

H. imperialis – H. sarrabusensis

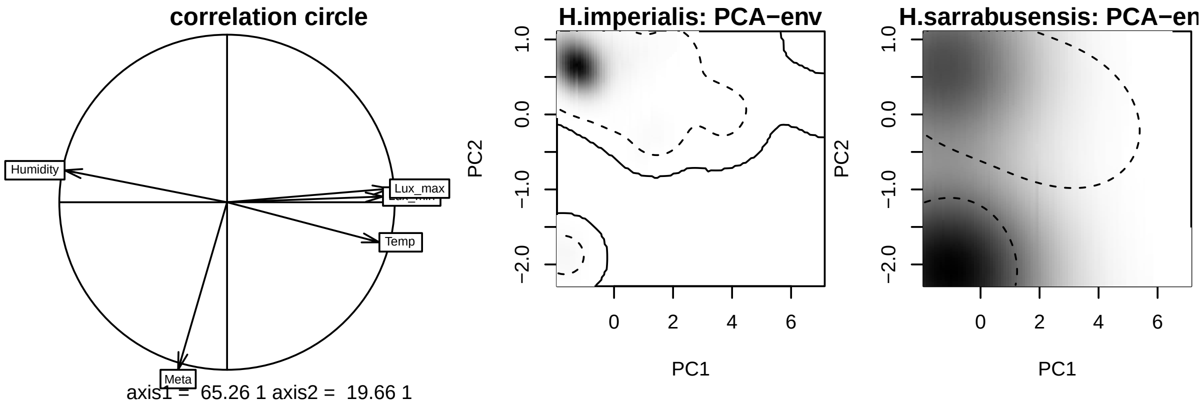

Fig. S2 (continues)

H. imperialis – H. strinatii

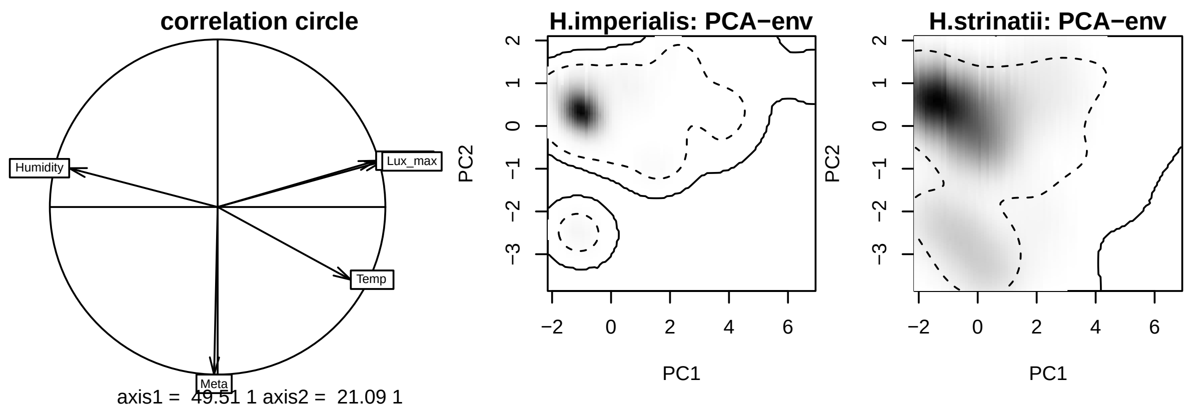

H. imperialis – H. supramontis

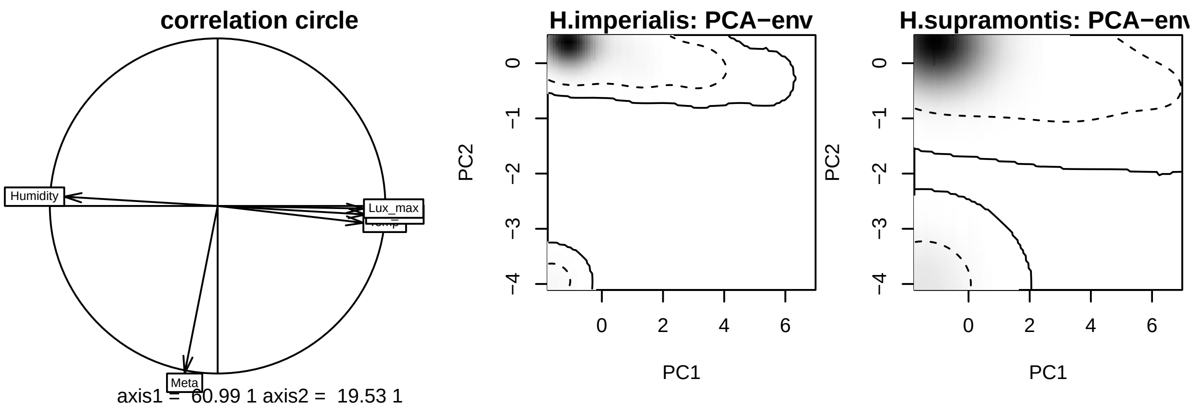

H. itlaicus – H. sarrabusensis

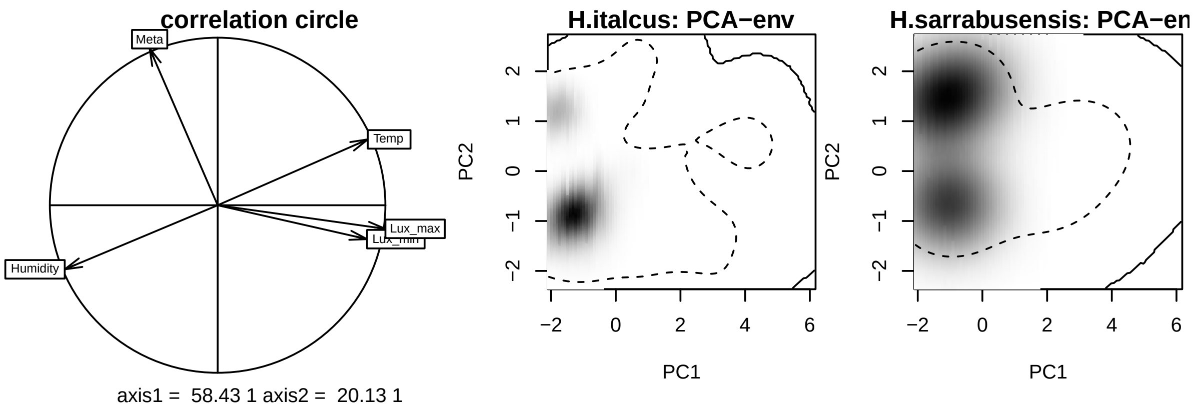

Fig. S2 (continues)

H. italicus – H. strinatii

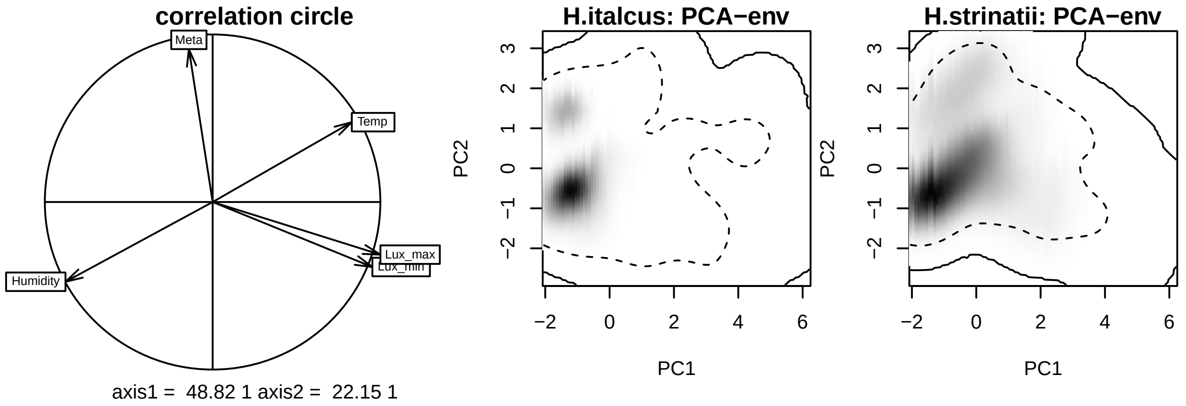

H. italicus – H. supramontis

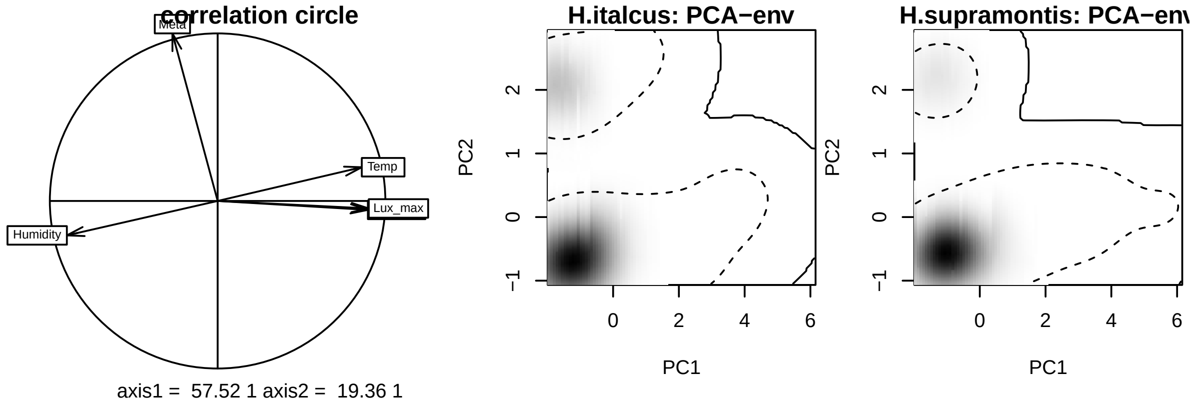

H. sarrabusensis – H. strinatii

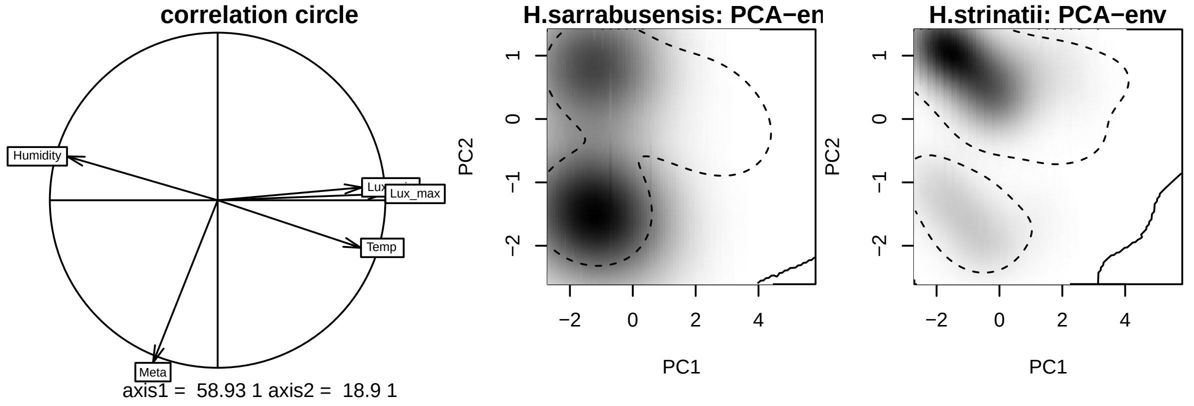

Fig. S2 (continues)

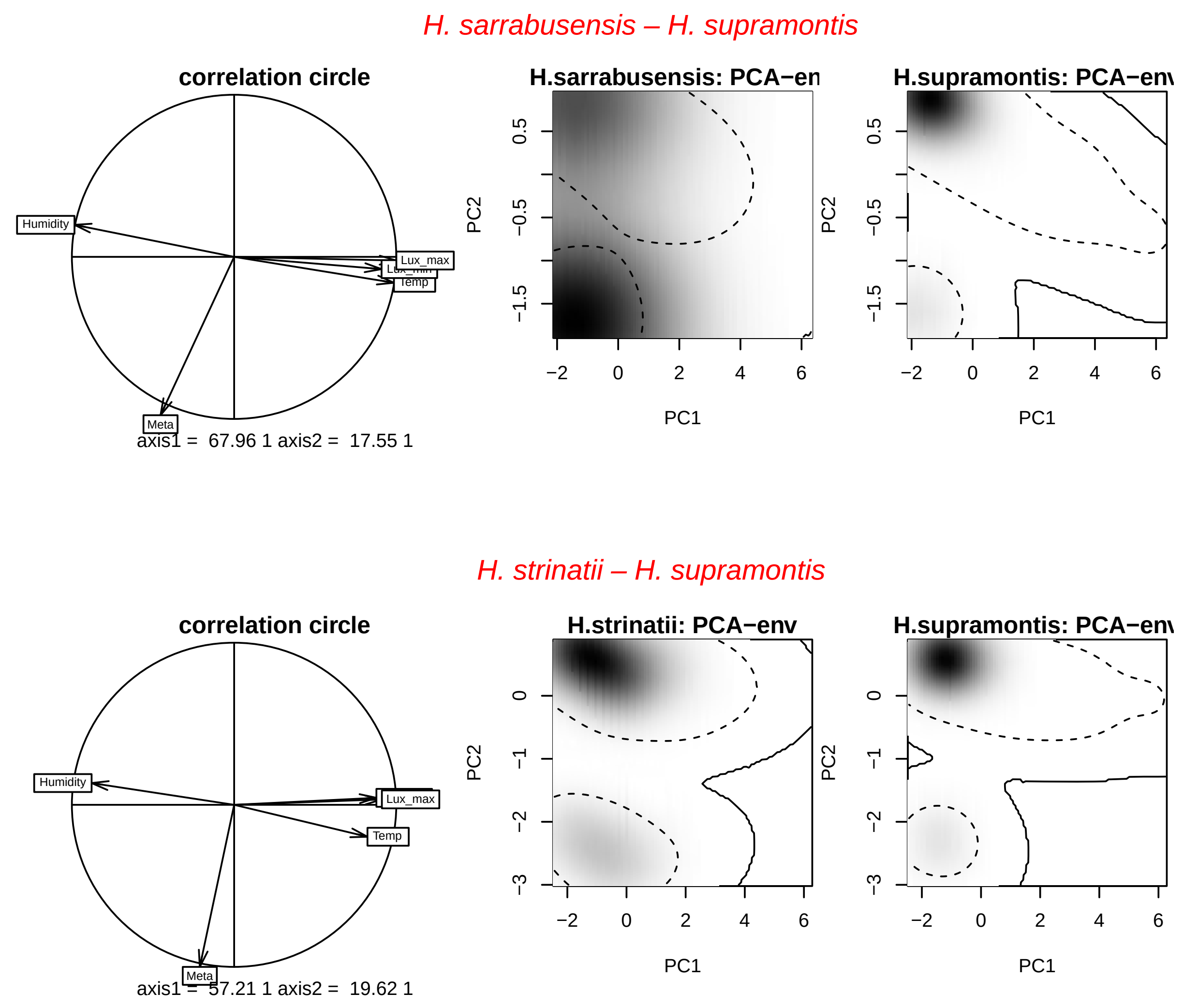
H. sarrabusensis – H. supramontis
correlation circle
Humidity
Lux_max
Temp
Meta
axis1 = 67.96 1 axis2 = 17.55 1
H.sarrabusensis: PCA−en
H.supramontis: PCA−env
PC1
PC2
H. strinatii – H. supramontis
correlation circle
Humidity
Lux_max
Temp
Meta
axis1 = 57.21 1 axis2 = 19.62 1
H.strinatii: PCA−env
H.supramontis: PCA−env
PC1
PC2

Fig. S2

Fig. S3. Results of PCA-env, representing the bioclimatic niche of the study species in the niche space. The left panels represent the contribution of the climatic variables on the PCA axes, and the percentage of inertia explained by the two axes. Central and right panels represent the niche of the species along the two first PCA axes. Grey shading shows the density of species occurrences by cell. The Shoener's *D* values and the results of similarity plots are reported in Table A3. Abbreviation of variables in the correlation circles are: Temp: mean temperature during the activity seasons (autumn, winter and spring); T_season: temperature seasonality; Prec: summed precipitation during the activity seasons; Prec_seas: precipitation seasonality; NDVI: Normalized Difference Vegetation Index.

H. ambrosii – H. flavus

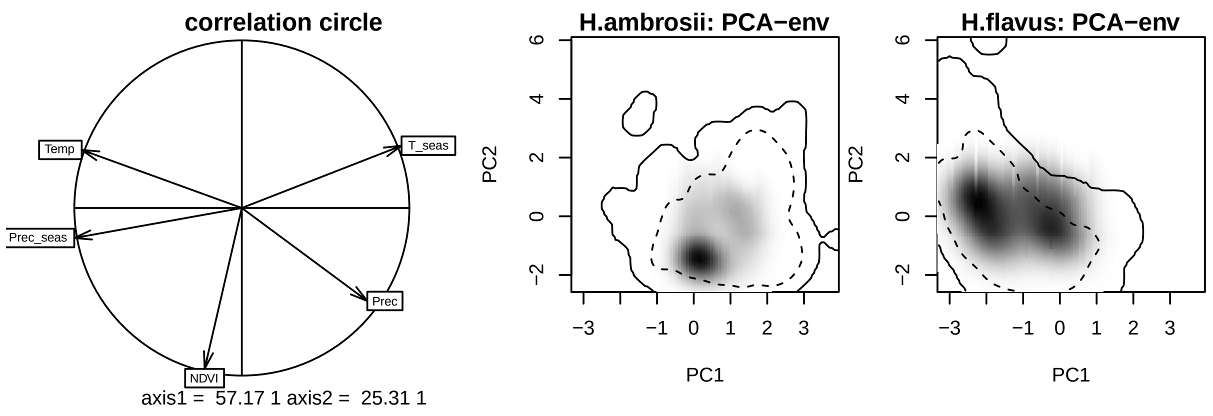

H. ambrosii – H. genei

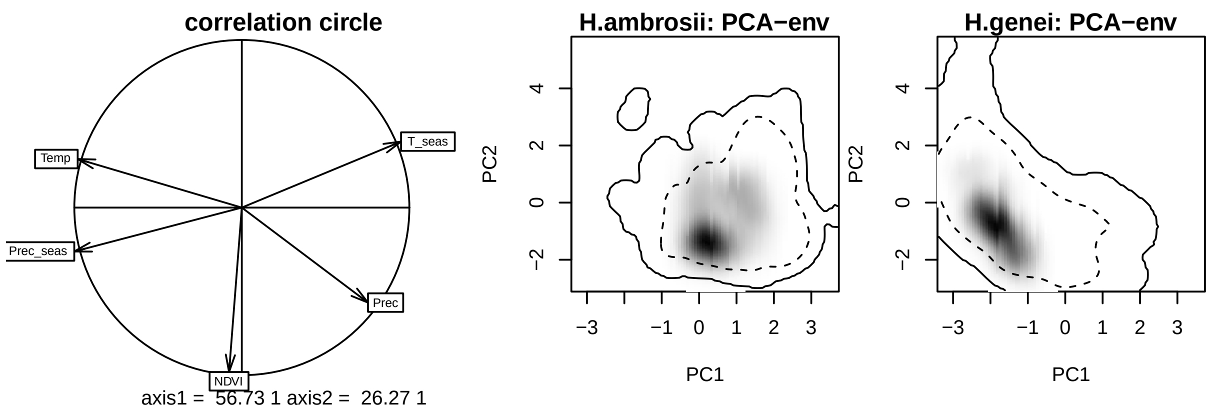

(continues)

H. ambrosii – H. imperialis

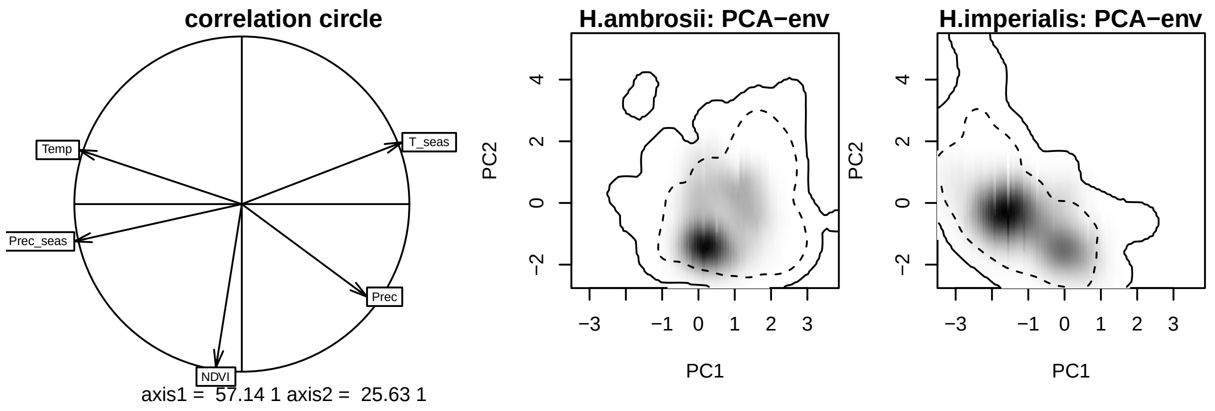

H. ambrosii – H. italicus

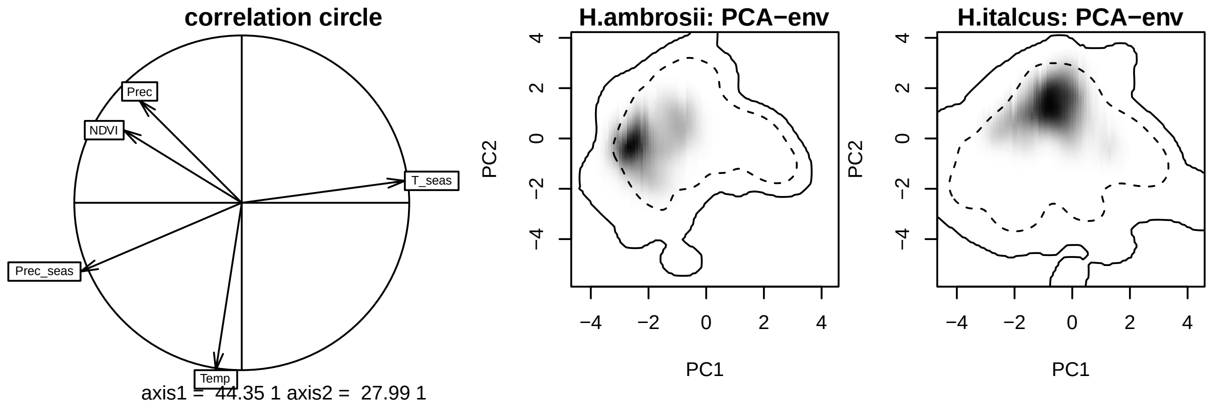

H. ambrosii – H. sarrabusensis

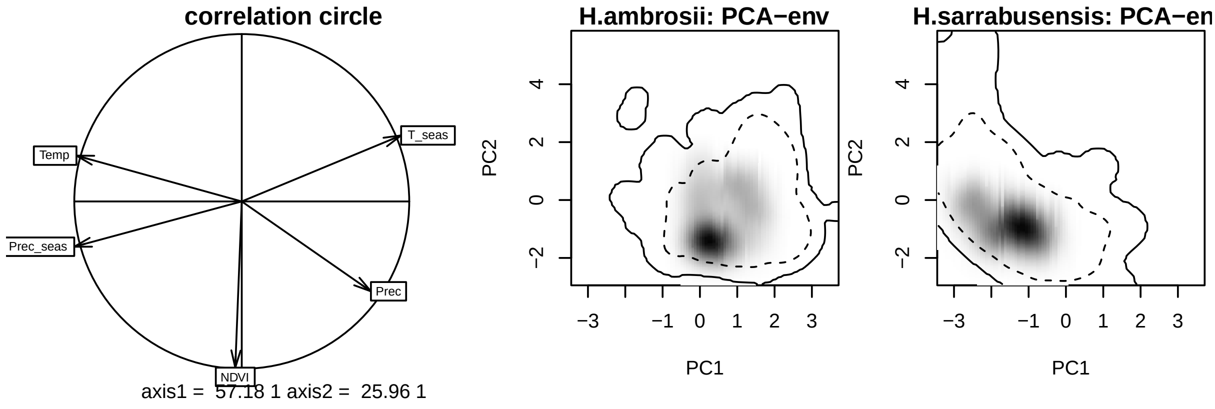

Fig. S3 (continues)

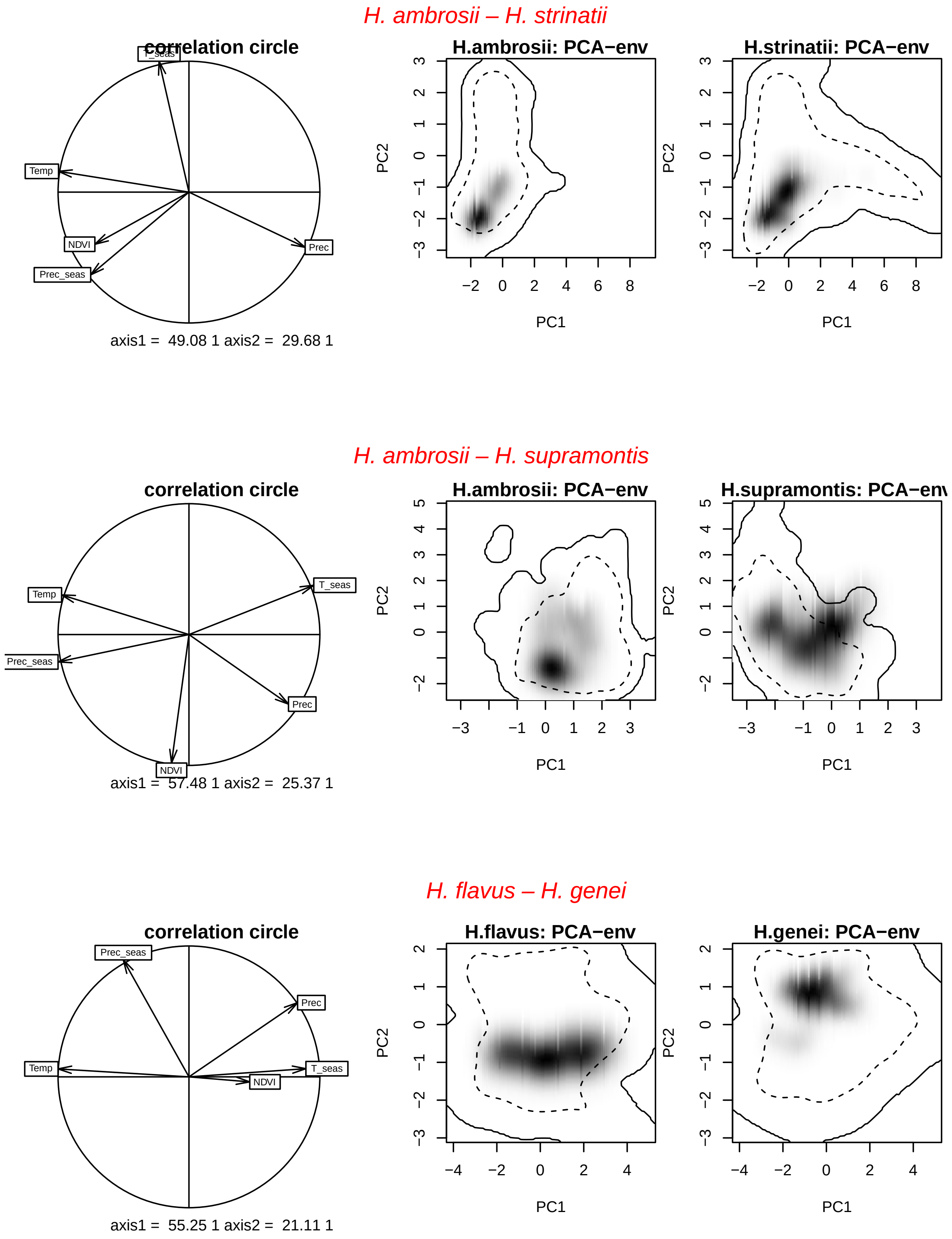

Fig. S3 (continues)

H. flavus – H. imperialis

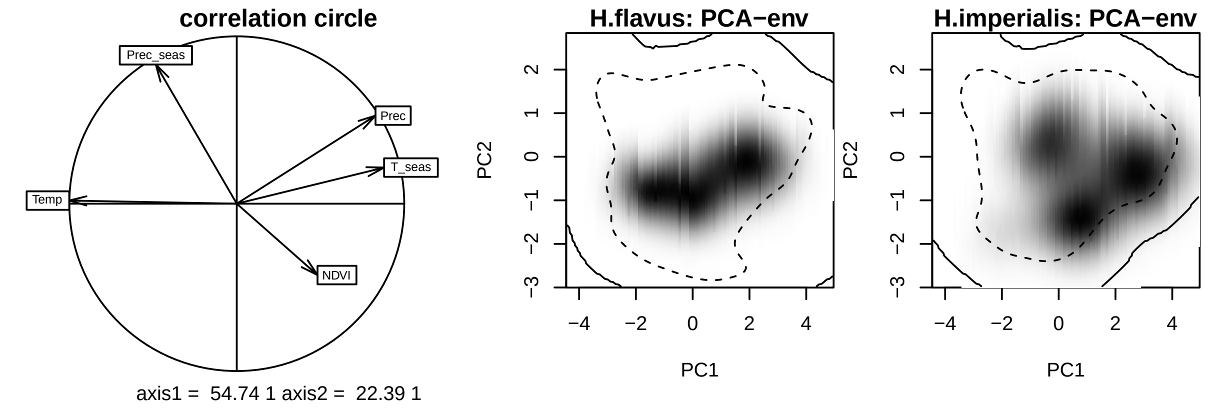

H. flavus – H. italicus

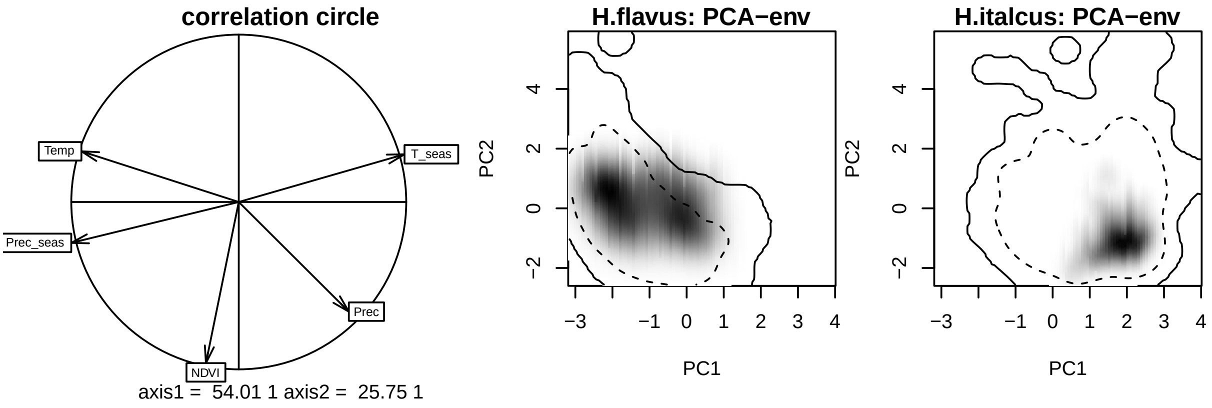

H. flavus – H. sarrabusensis

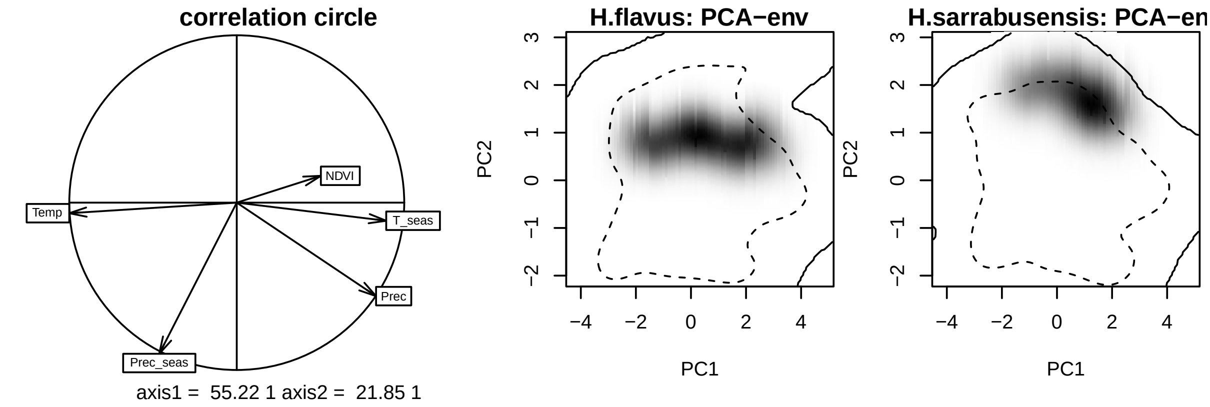

Fig. S3 (continues)

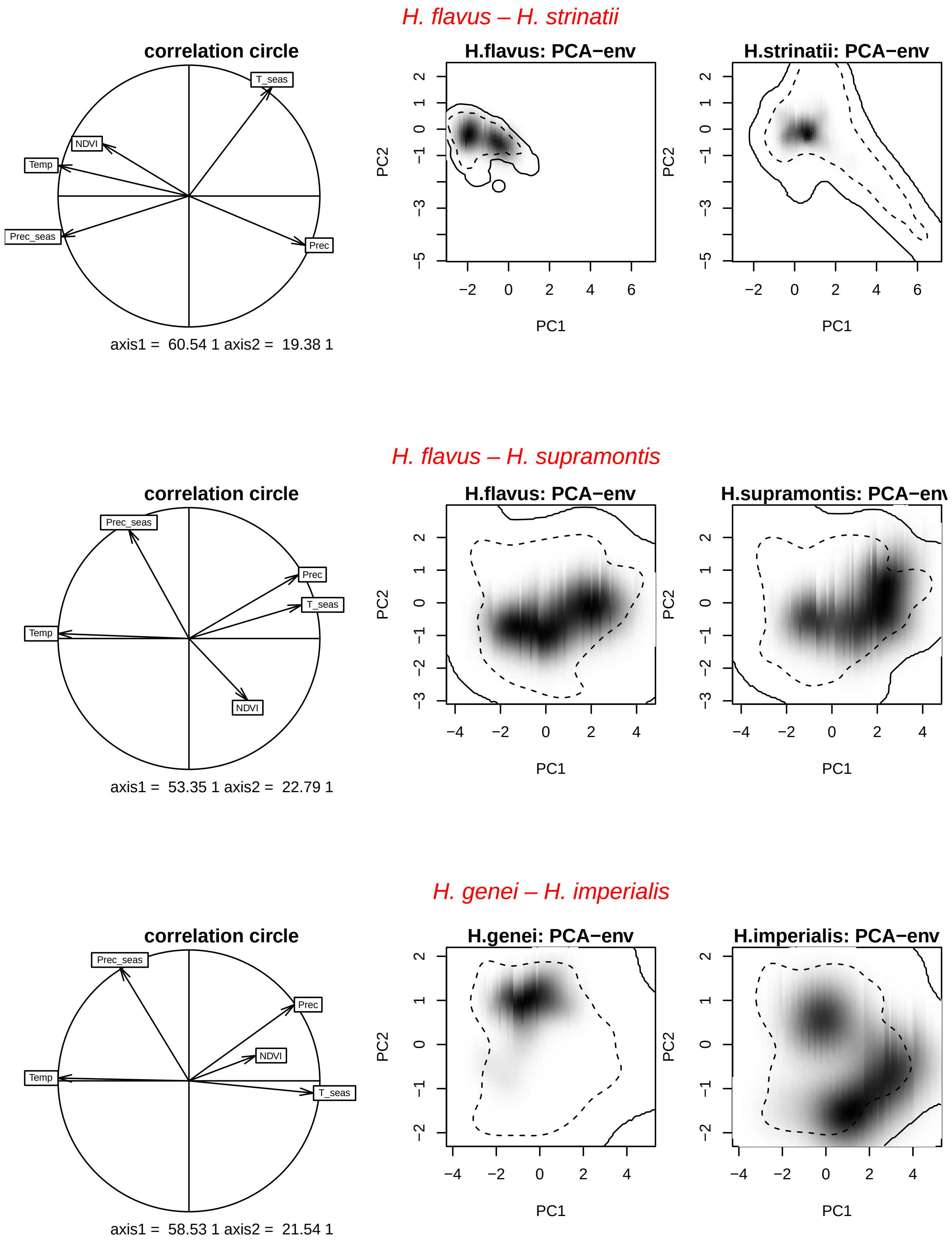

Fig. S3 (continues)

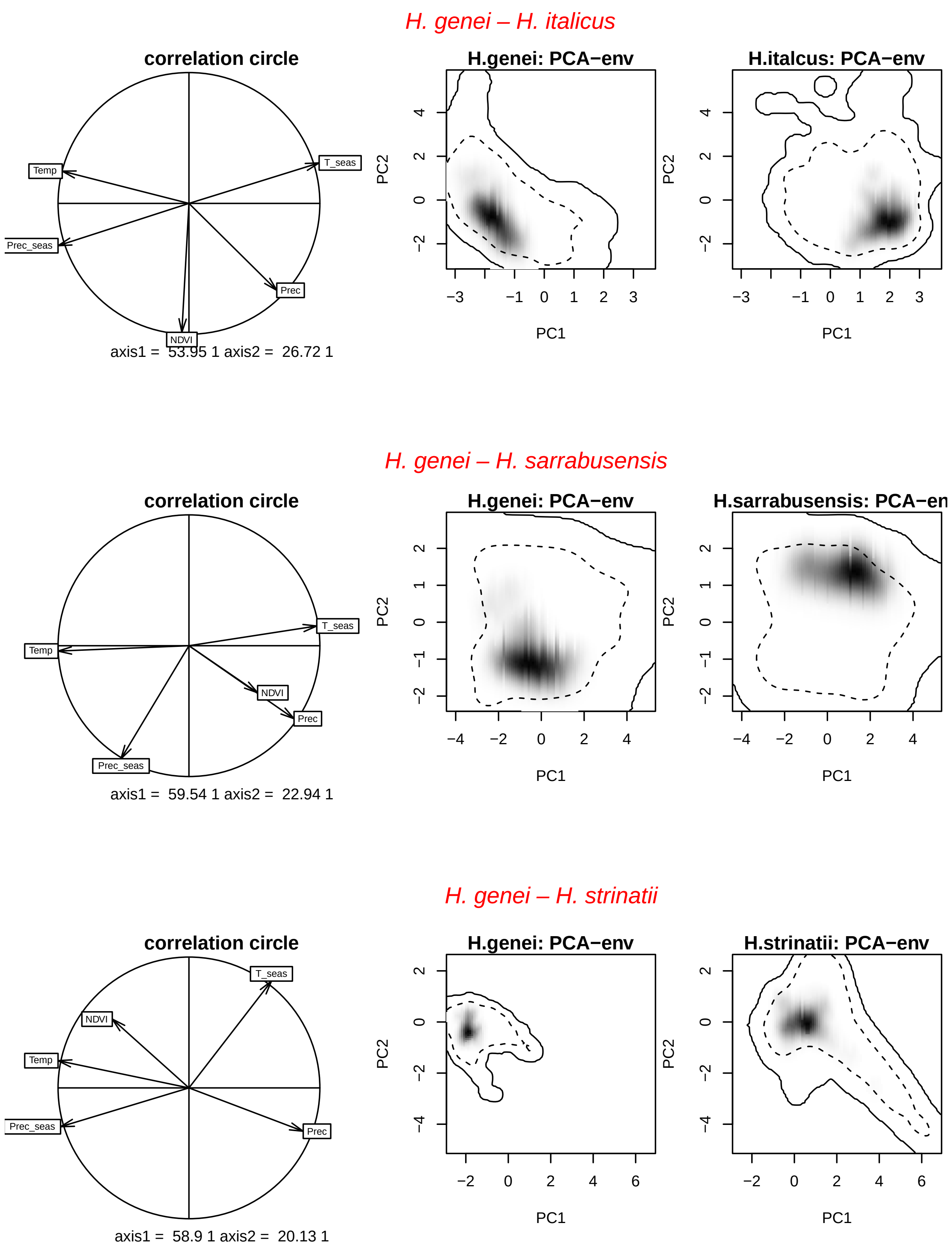

Fig. S3 (continues)

H. genei – H. supramontis

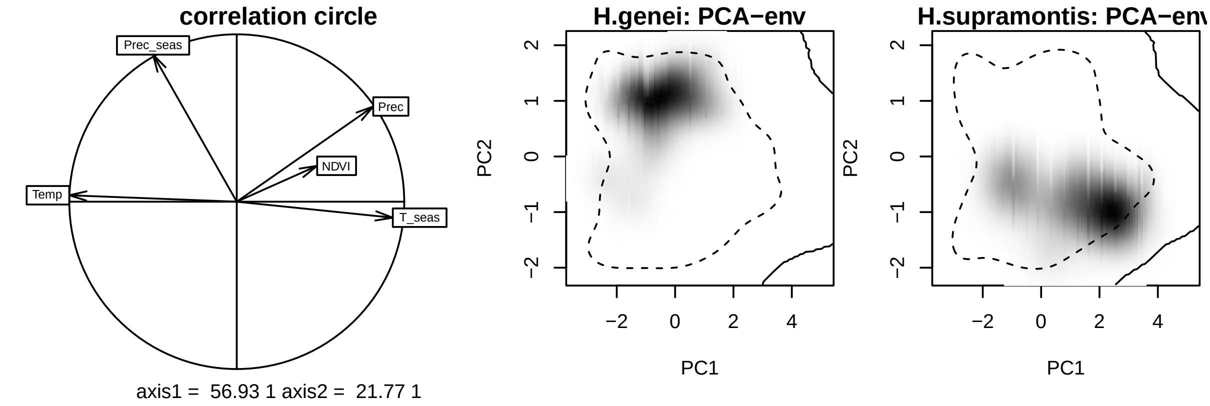

H. imperialis – H. italicus

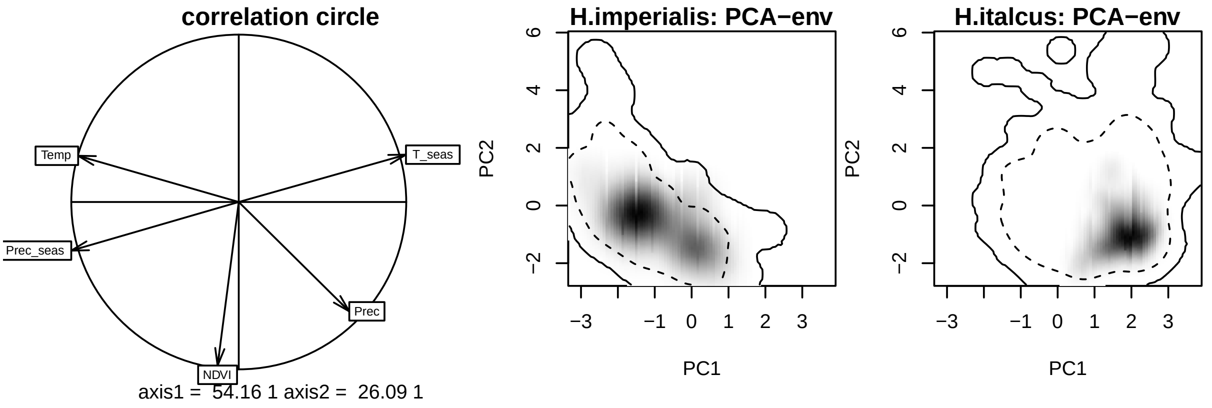

H. imperialis – H. sarrabusensis

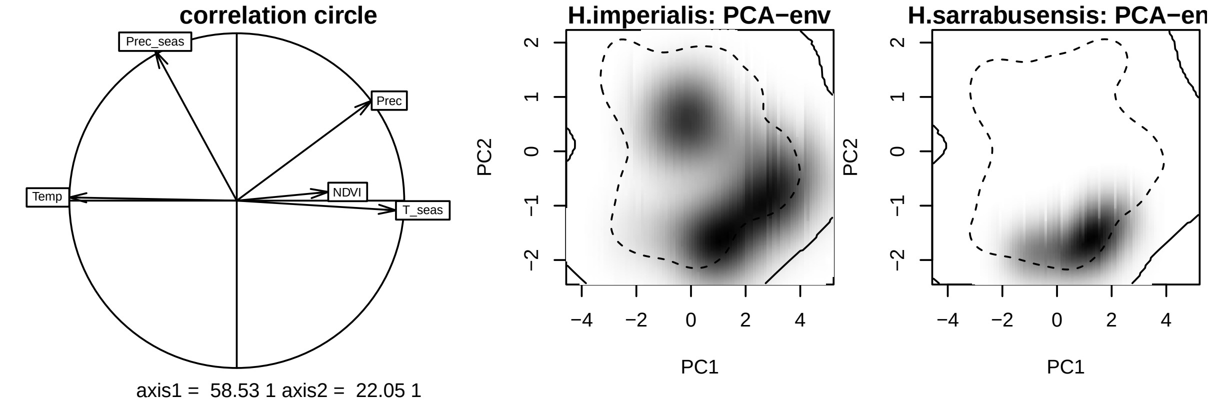

Fig. S3 (continues)

H. imperialis – H. strinatii

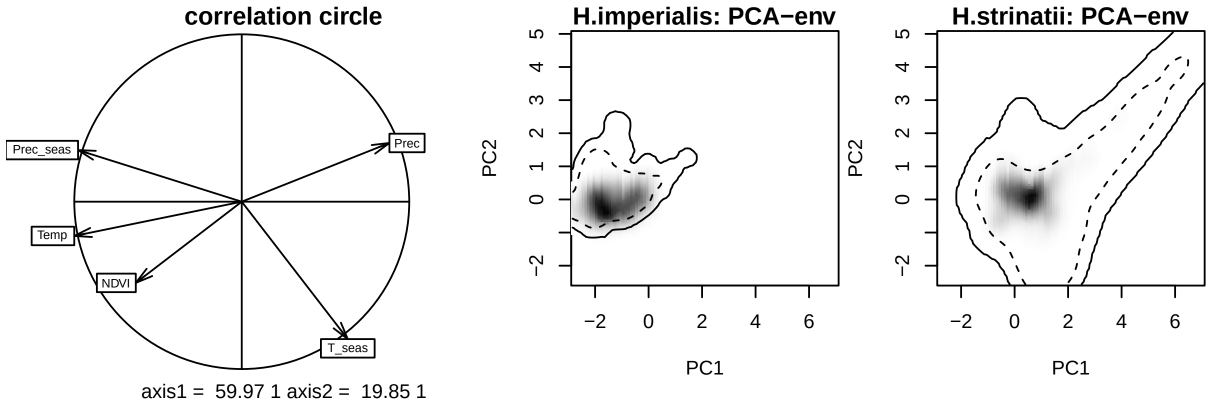

H. imperialis – H. supramontis

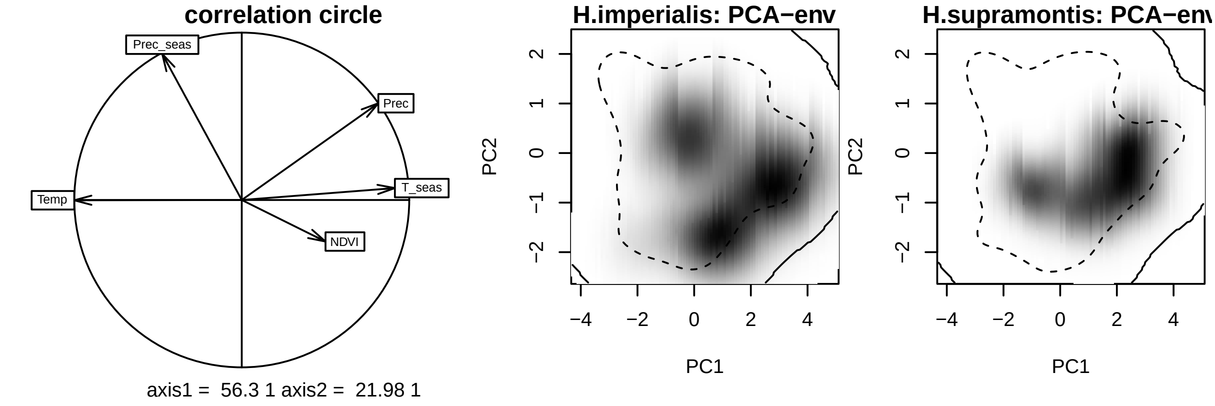

H. itlaicus – H. sarrabusensis

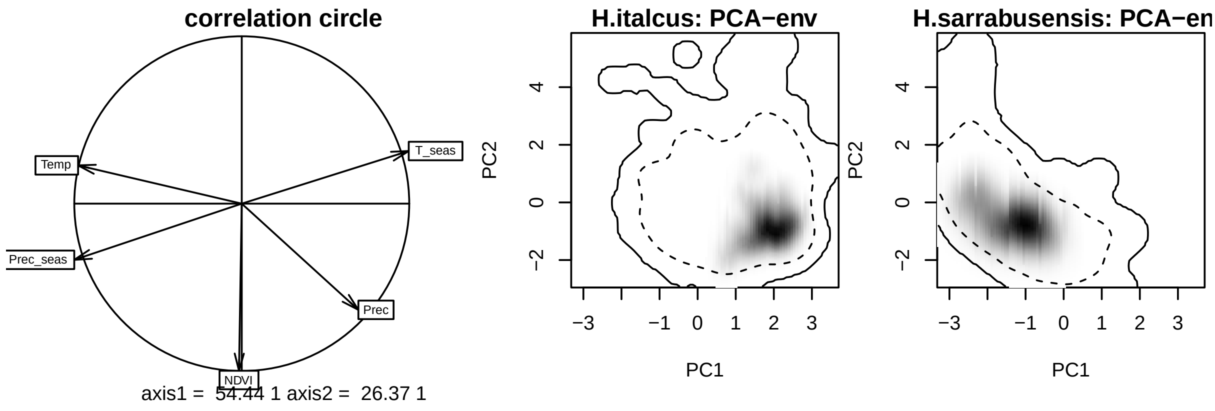

Fig. S3 (continues)

H. italicus – H. strinatii

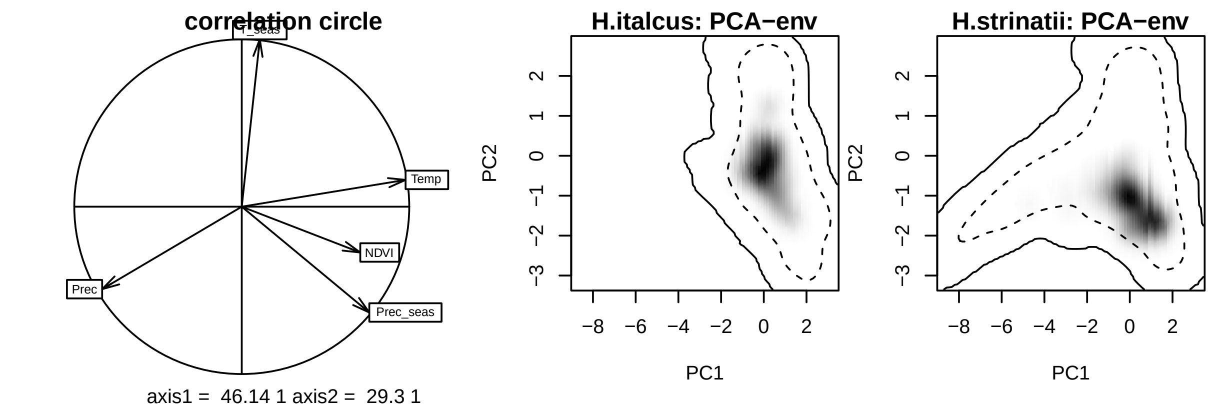

H. italicus – H. supramontis

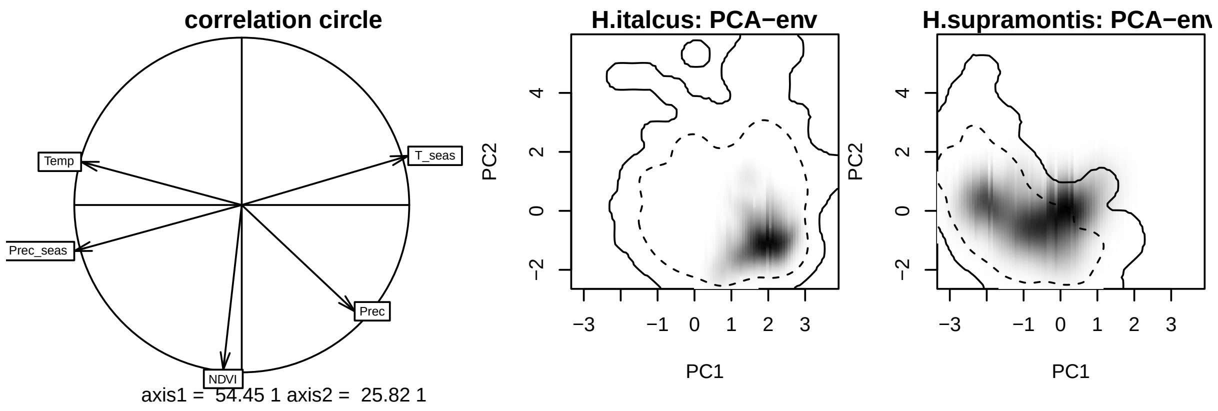

H. sarrabusensis – H. strinatii

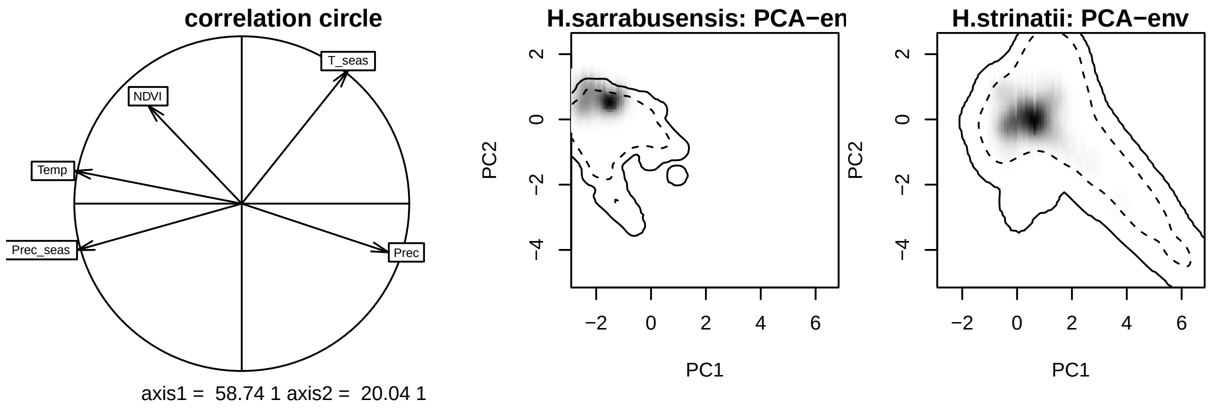

Fig. S3 (continues)

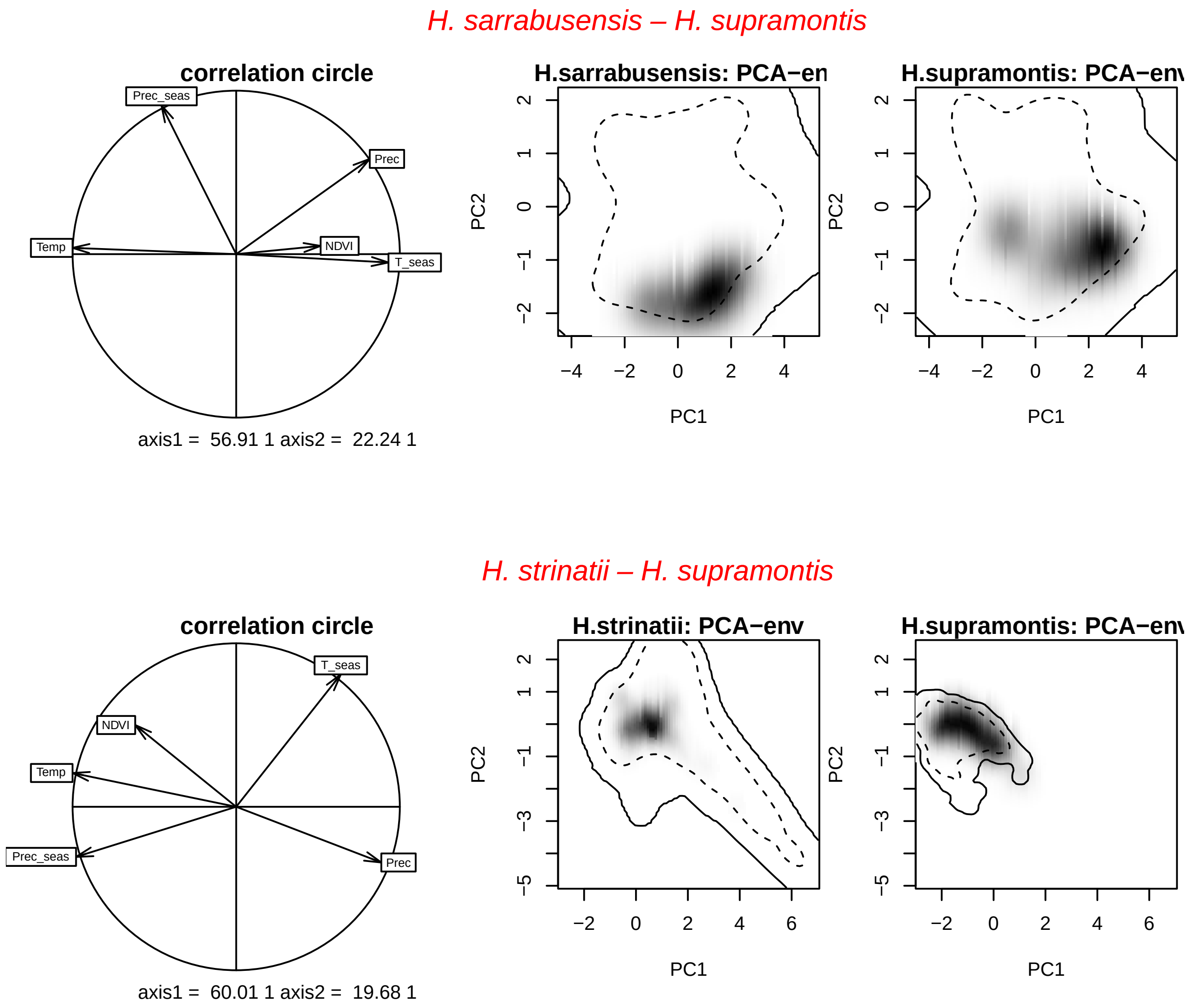

H. sarrabusensis – H. supramontis
correlation circle
Prec_seas
Prec
Temp
NDVI
T_seas
axis1 = 56.91 1 axis2 = 22.24 1
H.sarrabusensis: PCA−en
H.supramontis: PCA−env
PC1
PC2
H. strinatii – H. supramontis
correlation circle
T_seas
NDVI
Temp
Prec_seas
Prec
axis1 = 60.01 1 axis2 = 19.68 1
H.strinatii: PCA−env
H.supramontis: PCA−env
PC1
PC2

Fig. S3

Figure S4. Phylogenetic relationships between the eight European *Hydromantes* species. Relationships based on a molecular phylogeny combining mitochondrial and nuclear DNA sequence data. Redrawn from ref. [5].

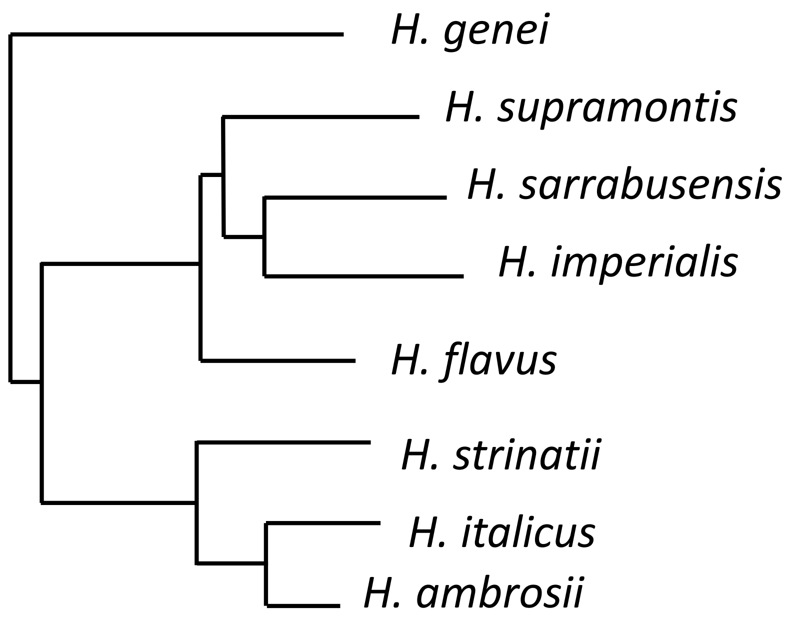

Figure S5. Niche differences among species according to a) microhabitat and b) bioclimatic analyses (multidimensional scaling plots), using annual temperature and precipitation instead of conditions during the activity seasons. Blue arrows are environmental variables added to plots using vector fitting.

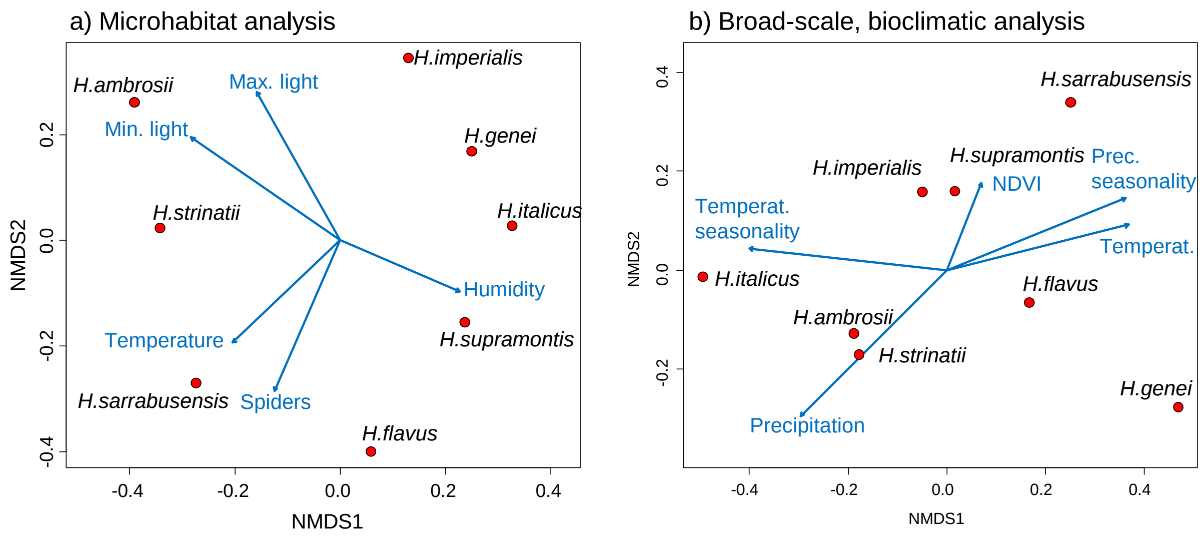

Figure S6. Niche differences among species according to bioclimatic analyses considering only presence points nearby localities used for microhabitat analyses: plot of multidimensional scaling. Procrustes rotation was used to allow comparison with Fig. 4. Blue arrows are environmental variables added to plots using vector fitting.

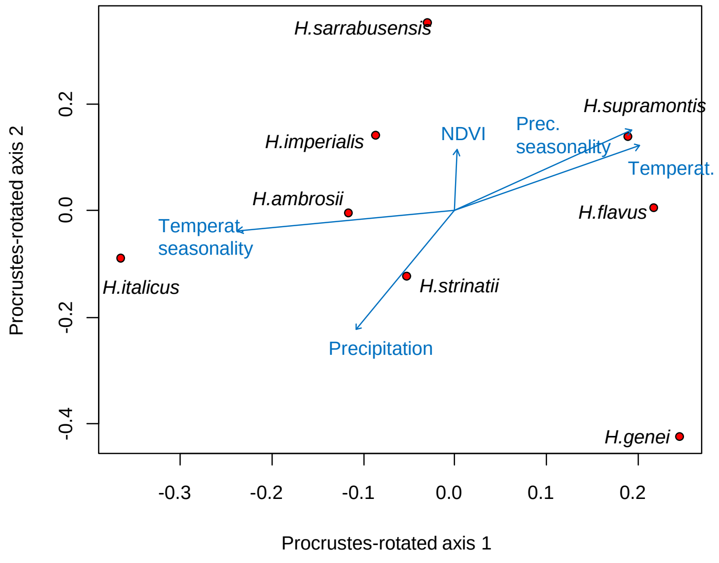

Figure S7. Relationship between operative temperature (mean temperature of sectors with active salamanders), and mean annual temperature measured on the basis of broad-scale variables.

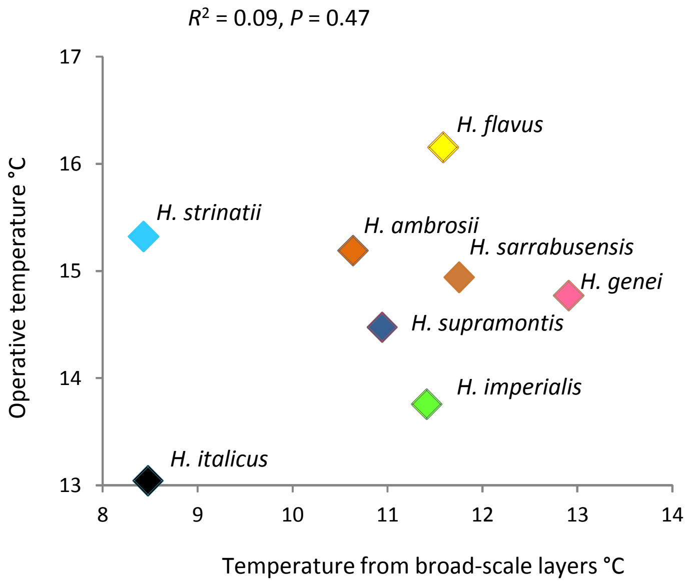

Figure S8. Relationships between air temperature measured inside the cave during surveys, and mean annual temperature (obtained from www.worldclim.org). a): Air temperature measured at 3m from cave entrance. b): Air temperature measured at 48m from cave entrance. The red line has slope = 1 and intercept = 0. At 3-m from the entrance, air temperature is often warmer than average annual temperature. Conversely, far from the entrance air temperature is strikingly similar to the average outdoor annual temperature, except in two outlier caves.

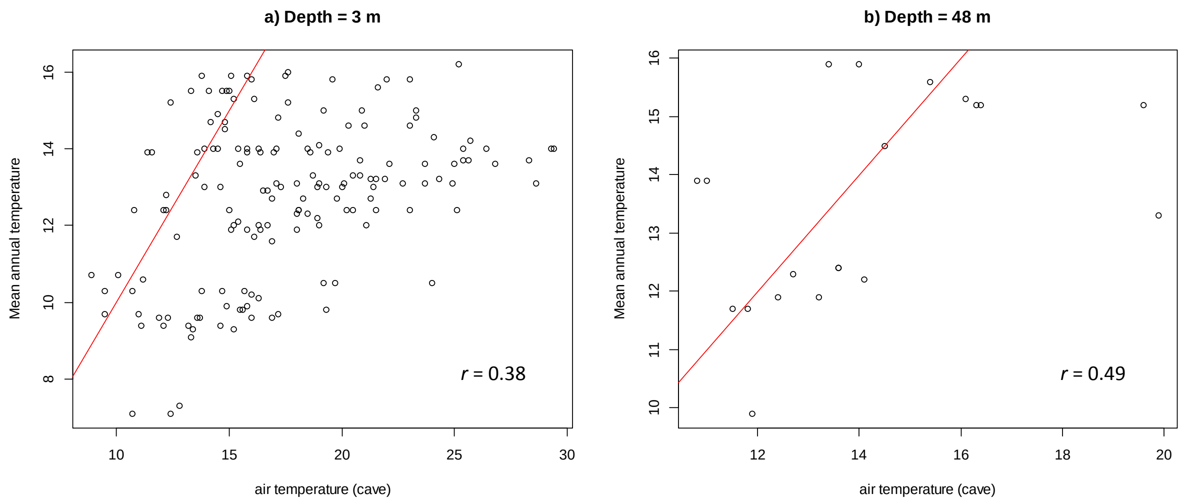

Supplement: Supplementary file 1 — Supporting Information [file 41598_2018_28796_MOESM1_ESM.pdf]
